# microRNA Expression Profile in Single Hormone Receptor-Positive Breast Cancers Is Mainly Dependent on HER2 Status—A Pilot Study

**DOI:** 10.3390/diagnostics10090617

**Published:** 2020-08-20

**Authors:** Michał Kunc, Marta Popęda, Anna Szałkowska, Magdalena Niemira, Michał Bieńkowski, Rafał Pęksa, Aleksandra Łacko, Barbara S. Radecka, Marcin Braun, Joanna Pikiel, Maria Litwiniuk, Katarzyna Pogoda, Ewa Iżycka-Świeszewska, Adam Krętowski, Anna J. Żaczek, Wojciech Biernat, Elżbieta Senkus-Konefka

**Affiliations:** 1 Department of Pathomorphology, Medical University of Gdansk, 80-214 Gdańsk, Poland; mkunc@gumed.edu.pl (M.K.); michal.bienkowski@gmail.com (M.B.); rafalpeksa@gumed.edu.pl (R.P.); biernat@gumed.edu.pl (W.B.); 2 Laboratory of Translational Oncology, Intercollegiate Faculty of Biotechnology, Medical University of Gdansk, 80-211 Gdansk, Poland; marta.popeda@gmail.com (M.P.); azaczek@gumed.edu.pl (A.J.Ż.); 3 Clinical Research Centre, Medical University of Bialystok, 15-276 Bialystok, Poland; anna.szalkowska@umb.edu.pl (A.S.); magdalena.niemira@umb.edu.pl (M.N.); adamkretowski@wp.pl (A.K.); 4 Department of Oncology, Wroclaw Medical University, 53-413 Wroclaw, Poland; olalacko@wp.pl; 5 Department of Oncology, Breast Unit, Lower Silesian Oncology Centre, 53-413 Wroclaw, Poland; 6 Department of Oncology, Institute of Medical Sciences, University of Opole, 45-052 Opole, Poland; brad@onkologia.opole.pl; 7 Department of Clinical Oncology, Tadeusz Koszarowski Cancer Center in Opole, 45-061 Opole, Poland; 8 Department of Pathology, Chair of Oncology, Medical University of Lodz, 92-213 Lodz, Poland; braunmarcin@gmail.com; 9 Department of Oncology, Szpital Morski, 81-519 Gdynia, Poland; joanna.pikiel@post.pl; 10 Department of Oncologic Pathology and Prophylaxis, Poznan University of Medical Sciences, 61-866 Poznan, Poland; maria@litwiniuk.net; 11 Department of Breast Cancer and Reconstructive Surgery, Maria Sklodowska Curie National Research Institute of Oncology, 02-781 Warsaw, Poland; katarzynapogoda@gazeta.pl; 12 Department of Pathology & Neuropathology, Medical University of Gdansk, 80-211 Gdansk, Poland; ewa.izycka-swieszewska@gumed.edu.pl; 13 Department of Oncology and Radiotherapy, Medical University of Gdansk, 80-214 Gdansk, Poland

**Keywords:** breast cancer, estrogen receptor, progesterone receptor, HER2, microRNA

## Abstract

Estrogen (ER) and progesterone (PgR) receptors and HER2 are crucial in the assessment of breast cancer specimens due to their prognostic and predictive significance. Single hormone receptor-positive breast cancers are less common and their clinical course is less favorable than ER(+)/PgR(+) tumors. Their molecular features, especially microRNA (miRNA) profiles, have not been investigated to date. Tumor specimens from 36 chemonaive breast cancer patients with known ER and PgR status (18 ER(+)/PgR(−) and 18 ER(−)/PgR(+) cases) were enrolled to the study. The expression of 829 miRNAs was evaluated with nCounter Human v3 miRNA expression Assay (NanoString). miRNAs differentiating between ER/PgR/HER2 phenotypes were selected based on fold change (FC) calculated for the mean normalized counts of each probe in compared groups. The differences were estimated with Student’s *t*-test or Two-Way ANOVA (considering also the HER2 status). The results were validated using The Cancer Genome Atlas (TCGA) dataset. Following quality control of raw data, fourcases were excluded due to low sample quality, leaving 14 ER(+)/PgR(−) and 18 ER(−)/PgR(+) cases. After correction for multiple comparisons, we did not find miRNA signature differentiating between ER(−)/PgR(+) and ER(+)/PgR(−) breast cancers. However, a trend for differing expression (*p*-value ≤ 0.05; FDR > 0.2; ANOVA) in eight miRNAs was observed. The ER(+)/PgR(−) group demonstrated elevated levels of four miRNAs—miR-30a-5p, miR-29c-3p, miR-141-3p and miR-423-5p—while the ER(−)/PgR(+) tumors were enriched in another four miRNAs—miR-514b-5p, miR-424-5p, miR-495-3p, and miR-92a-3p. For one of the miRNAs—miR-29c-3p—the association with the ER(+)/PgR(−) phenotype was confirmed in the TCGA cohort (*p*-value = 0.024; *t*-test). HER2 amplification/overexpression in the NanoString cohort was related to significant differences observed in 33 miRNA expression levels (FDR ≤ 0.2; ANOVA). The association with HER2 status was confirmed in the TCGA cohort for four miRNAs (miR-1180-3p, miR-223-3p, miR-30d-5p, and miR-195-5p). The main differences in miRNA expression amongst single hormone receptor-positive tumors were identified according to their HER2 status. However, ER(+)/PgR(−) cases tended to express higher levels of miRNAs associated with ER-positivity (miR-30a-5p, miR-29c-3p, miR-141-3p), whereas ER(−)/PgR(+) cancers showed elevated levels of miRNAs characteristic for double- and triple-negative tumors (miR-92a-3p, miR-424-5p). Further studies are necessary to comprehensively analyze miRNA signatures characteristic of ER(−)/PgR(+) and ER(+)/PgR(−) tumors.

## 1. Introduction

Breast cancer is the most frequent malignancy and the most common cause of cancer-related death in women worldwide. The expression of estrogen receptor (ER), progesterone receptor (PgR), and HER2 are crucial in the assessment of breast cancer specimens due to their prognostic and predictive significance. PgR expression in the mammary gland is dependent on ER, thus, these two receptors are usually co-expressed [1]. In 15% of cases, PgR expression is lost in ER(+) cancers, whereas a lack of nuclear ER expression in PgR(+) tumors is unusual. ER(+)/PgR(−) tumors tend to present less favorable clinicopathological features and a higher risk of relapse than ER+/PgR+ cancers [2,3]. Loss of PgR expression may be related to various mechanisms, including nonfunctional ER, epigenetic modifications of PgR promoter, low levels of circulating estrogens, and altered ER co-regulators [4]. The existence of ER(−)/PgR(+) tumors has been questioned and mostly regarded as an artifact in immunohistochemical staining. Nevertheless, they are still encountered in practice; our own experience and thorough literature analysis indicate that at least some cases of ER(−)/PgR(+) tumors are non-artifactual [1,5] but are characterized by a unique clinical course and biological features, including high-grade histology and the prognosis being an intermediate between triple-negative and double-positive tumors.

MicroRNAs (miRNAs) are short non-coding RNAs, which regulate gene expression. They are transcribed by RNA polymerase II. Precursor forms of miRNAs are processed by endoribonuclease Dicer in the cytoplasm. Subsequently, they are incorporated into RNA-induced silencing complex (RISC) and modulate expression of genes via mRNA cleavage and degradation or translational repression [6].

miRNAs have multiple roles in cancer biology as they may serve as tumor suppressors and oncogenes (tsmiRs and oncomiRs, respectively) [7]. Nevertheless, their significance is much broader, since they regulate cancer cell metabolism and host immune response and expression of potentially targetable proteins [8]. In neoplastic cells, some miRNAs are upregulated, whereas others are downregulated, thus their differential expression may potentially serve as diagnostic, prognostic, and predictive markers in various malignancies, including breast cancer [9].

miRNA expression profile of single hormone receptor-positive tumors has been poorly investigated so far. However, studies focused on ER+/PgR+ breast cancer indicate that miRNAs interact reciprocally with ER and PgR receptors [10]. Recently, small RNA sequencing of 186 tumor samples showed that miRNA expression can be translated into intrinsic molecular subtypes of breast cancer [11]. The cluster consisting of miR-99a/let-7c/miR-125b miRNA separated luminal A and luminal B subtypes, whereas miR-4728 was a specific marker of the HER2-enriched subgroup.

In the current pilot study we aimed to identify differentially expressed miRNAs in two types of single hormone receptor-positive breast cancers (ER(+)/PgR(−) and ER(−)/PgR(+)) with further distinction into HER2-overexpressing/amplified and HER2-negative tumors in a well-established cohort collected at the Medical University of Gdańsk. Owing to the complexity of breast cancer and the essential role of both HER2 and hormone receptors (ER and PgR) in its biology, we were interested in evaluating the differences in miRNA profiles between the four ER/PgR/HER2 phenotypes of single hormone receptor-positive primary breast tumors. To validate the results we used a publicly available dataset from The Cancer Genome Atlas Project (TCGA, https://www.cancer.gov/tcga).

## 2. Materials and Methods

### 2.1. Study Group

A total of 96 breast cancer patients diagnosed with a confirmed single hormone receptor-positive tumor (64 ER(+)/PgR(−) and 32 ER(−)/PgR(+)) were screened for eligibility. The patients were diagnosed in 9 Polish centers (Medical University of Gdańsk; Lower Silesian Oncology Center, Wrocław; Tadeusz Koszarowski Regional Oncology Center, Opole; Medical University of Łódź; Gdynia Maritime Hospital; Greater Poland Cancer Centre, Poznań; The Maria Skłodowska-Curie National Research Institute of Oncology, Warsaw; Beskid Oncology Center, Bielsko-Biała; Copernicus Hospital, Gdańsk) between 2007 and 2018.

All cases underwent central review by pathologists experienced in breast cancer (R.P. and M.K.) to confirm the diagnosis and receptor status. Three antibodies against ER were utilized (Dako monoclonal (MC) mouse anti-ERα, clone 1D5; Dako MC rabbit anti-ERα, clone EP1; VENTANA Roche MC rabbit anti-ERα, clone SP1), and one against PgR (Dako MC mouse anti-PgR, clone 636). Only cases with < 1% stained tumor nuclei were regarded as negative for a given receptor according to American Society of Clinical Oncology/College of American Pathologists (ASCO/CAP) criteria (Hammond et al. 2010). Histologically normal breast epithelium adjacent to carcinoma was used as an internal positive control. HER2 status was routinely evaluated by immunohistochemistry and/or by hybridization in situ and was obtained from the medical records. Subsequently, propensity score matching was performed using the Matching package [12] according to age, grade, HER2 status, and Ki67 status. Only cases with a sufficient amount of tumor tissue for molecular testing were enrolled. Thus, 36 paired single hormone receptor-positive cases were included (18 ER(+)/PgR(−) and 18 ER(−)/PgR(+)). The study was approved by the Bioethical Committee of the coordinating center, Medical University of Gdansk, Poland (approval no: NKBBN/119/2018; 10 April 2018). All research was performed in accordance with the appropriate regulations.

### 2.2. NanoString nCounter Assay for miRNA Profiling

Total RNA, including miRNA, was isolated from archival FFPE blocks (four 20 μm-thick sections per block) using RecoverAll™ Total Nucleic Acid Isolation Kit for FFPE (Invitrogen, Carlsbad, CA, USA) following the manufacturers’ protocol. RNA concentration and purity were determined using a NanoDrop 1000 spectrophotometer (Thermo Scientific, Waltham, MA, USA).

Extracted RNA (3 μl) was subjected to miRNA expression profiling with nCounter Human v3 miRNA Expression Assay (NanoString Technologies, Seattle, WA, USA) according to the manufacturer’s procedures for hybridization, detection, and scanning [13]. Raw NanoString expression data were submitted to the GEO database under GSE155362 accession number.

Following quality control of raw data, 4 cases were excluded from analysis due to low sample quality and resulting ligation issues, thus the final study group counted 14 ER(+)/PgR(−) and 18 ER(−)/PgR(+) cases (characterised in Table 1).

For each analyzed sample, correction and normalization were performed using nSolver 4.0 software, as previously described [14]. In brief, the background level was estimated by thresholding over the mean plus 2 standard deviations of the negative control counts. Subsequently, the data were normalized according to the global mean of the counts of positive controls and all miRNA genes. The negative and positive control probes were included in the assay.

Transcripts detected in <1/3 of the whole NanoString group (<10 cases) were excluded, leaving 185 out of 798 miRNAs for further analysis.

### 2.3. The Cancer Genome Atlas (TCGA) miRNA Data Processing

Clinical and miRNA-seq data of the Breast invasive carcinoma (BRCA) cohort were obtained from the TCGA portal (data status of 28 January 2016). The methods of biospecimen procurement, RNA isolation, and RNA sequencing were previously described by The Cancer Genome Atlas Research Network [15,16].

The Illumina HiSeq miRNA-seq dataset (illuminahiseq_mirnaseq-miR_gene_expression), covering normalized counts of sequences aligning to 1046 miRNA transcripts (“reads_per_million_miRNA_mapped”) in 756 primary breast tumors, were selected for analysis. Records with missing clinical or expression values were excluded. The group was limited to single hormone receptor-positive tumors—ER(+)/PgR(−) and ER(−)/PgR(+)—from female patients, not exposed to neoadjuvant systemic therapy, with known HER2 status, leaving 67 out of 756 cases for further analysis (characterized in Table 2 and listed in Supplementary Table S1). The overlap between our series (further referred to also as the NanoString group; miRBase version 21) and the TCGA dataset (miRBase version 16) was determined using the miRBaseConverter package [17]. Two hundred and twelve (212) miRNAs were assessed in both NanoString and TCGA data. Due to the unbalanced proportions of ER/PgR/HER2 subgroups in the TCGA cohort (reflecting the population frequency), the power of the analysis was limited, which precluded the screening of all miRNAs. Thus, only the miRNAs differing between subgroups in the NanoString data were investigated in the TCGA dataset (the chart of data processing is shown in Supplementary Figure S1 and a list of miRNA analysis is given in Supplementary Table S2).

### 2.4. miRNA Targets Prediction and Functional Annotation

miRNET 2.0 database including miRTarBase 8.0 (www.mirnet.ca) was employed to identify target genes of selected miRNAs in mammary gland tissue [18]. The experimentally confirmed targets were subjected to functional annotation analysis (Gene Ontology biological processes (GO BP) using the Functional Annotation Tool by DAVID Bioinformatics Resources 6.81) [19,20].

### 2.5. The Cancer Genome Atlas (TCGA) mRNA Data Processing

mRNA-seq data (RNASeqV2, RSEM_ normalized), covering normalized counts of sequences aligning to 20,531 mRNA transcripts in 1091 primary breast tumors, were obtained from the TCGA portal (data status of 28 January 2016). Records with missing clinical or expression values were excluded. The group was limited to tumors with known hormone receptor status (ER and PgR) from female patients not exposed to neoadjuvant systemic therapy, leaving 1012 out of 1091 cases for further analysis (listed in Supplementary Table S1). The distribution of ER/PgR phenotypes in the group was as follows: ER(−)/PgR(-)—218 (22%), ER(−)/PgR(+)—16 (2%), ER(+)/PgR(−)—117 (12%), ER(+)/PgR(+)—661 (65%).

Analysis of reciprocal miRNA-mRNA expression was performed on the Illumina HiSeq sub-cohort of single hormone receptor-positive patients (*n* = 67) with miRNA profiling data available. For each of the top20 GO BP terms enriched in miRNA targets identified in the NanoString cohort, mRNA targets of ER/PgR-associated miRNAs (5/8 available in TCGA dataset) were extracted and their expression was correlated with the targeting miRNAs. For GO BP terms with at least 5 mRNA targets for each miRNA, the overlap of correlated mRNAs (cor > 0.3 or cor ≤ −0.3; Pearson’s method) between miRNAs was illustrated with Venn diagrams [21].

Analysis of unique mRNA-differentiating single hormone receptor-positive tumors from other phenotypes, or the single hormone receptor-positive tumors from each other (ER(+)/PgR(−) vs ER(−)/PgR(+)), was performed on the whole TCGA mRNA-seq cohort (*n* = 1012). For each mRNA transcript, differences in expression between compared phenotypes were reported as log2FC and estimated using *t*-test with Benjamini–Hochberg correction for multiple testing. Genes with log2FC > 1 or log2FC ≤ 1 and FDR > 0.05 were classified as differentiating between the compared phenotypes. Unique differentiating genes were identified via Venn diagram-based analysis of overlap between the lists generated for all compared groups.

## 3. Statistical Analysis

The data were analyzed using the R statistical environment (3.6.1) [22]. miRNAs differentiating between ER/PgR/HER2 phenotypes were selected based on logarithmic fold change (log2FC) calculated for the mean normalized counts of each probe in compared groups. miRNAs with log2FC ≥ 0.3 were considered upregulated; miRNAs with log2FC < −0.3 were considered downregulated. The differences were estimated with Student’s *t*-test (for ER/PgR and HER2(+)/HER2(−) comparisons) or Two-Way ANOVA (for ER/PgR/HER2 comparisons) with Benjamini–Hochberg correction for multiple testing. Differences in distribution of categorical variables between groups (clinicopathological characteristics) were estimated using Fisher’s Exact test. Correlation between linear variables (miRNA and mRNA expression) was estimated using Pearson’s method. *p*-values ≤ 0.05 and false discovery rate (FDR) values ≤ 0.2 were considered statistically significant.

Propensity score matching for ER(+)/PgR(−) and ER(−)/PgR(+) groups was performed using the Matching package [12]. The overlap between NanoString dataset (miRBase version 21) and the TCGA dataset (miRBase version 16) was determined using the miRBaseConverter package [17]. Heatmap was generated using heatmap3 package [18] and Venn diagrams were generated using venn package [21].

## 4. Results

### 4.1. Comparison of Study Groups

ER(−)/PgR(+) tumors were characterized by a higher grade and a higher Ki-67 index than ER(+)/PgR(−) cancers in the NanoString cohort, whereas in the TCGA cohort, ER(+)/PgR(−) patients presented more frequently with positive lymph nodes (Table 1 and Table 2). When compared to the TCGA cohort, our group was overrepresented by T1 tumors, and HER2-overexpressing/amplified cases (Supplementary Figure S2).

### 4.2. miRNA Expression Profile Associated with HER2 Status

**Figure 1:**
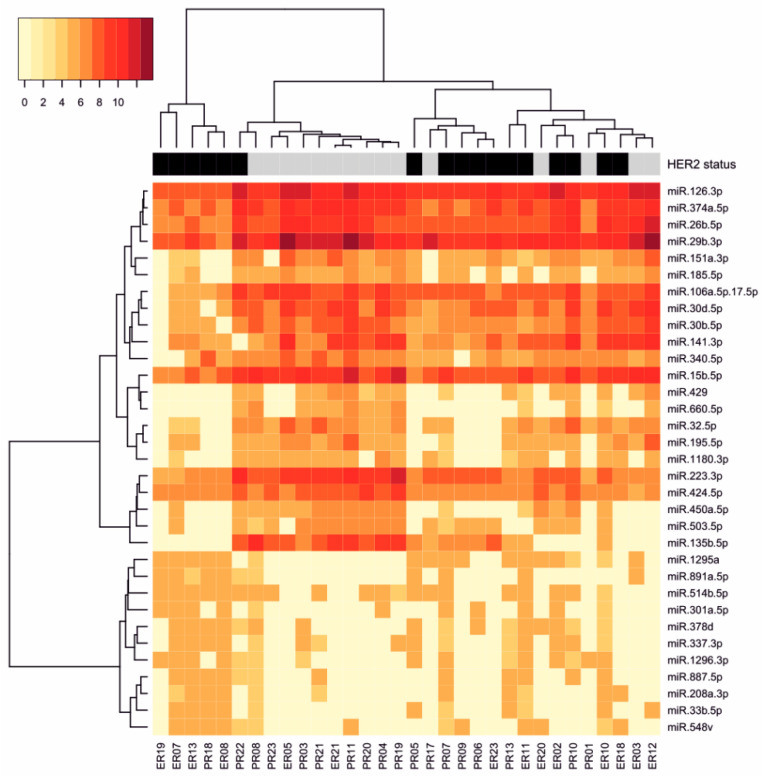
Heatmap depicting expression of 33 HER2-associated miRNAs among analyzed single hormone receptor-positive breast tumors; color legend indicates HER2 status; HER2-negative cases are marked in grey, HER2-positive cases are marked in black.

HER2 amplification/overexpression was related to significant differences observed in 33 miRNA expression levels (FDR ≤ 0.2; ANOVA). Eleven miRNAs were overexpressed and 22 miRNAs were under-expressed in HER2-positive cancers when compared to HER2-negative cancers (Table 3, Supplementary Figure S3). The most upregulated was miR-887-5p (FC 7.21), while miR-660-5p was the most downregulated (FC 0.20). Differentially expressed miRNAs are represented with a heatmap visualization (Figure 1), and with a volcano plot (Supplementary Figure S4).

These results were partially validated in the TCGA cohort (23/33 available for analysis), confirming HER2-related downregulation of four miRNAs from our cohort (miR-30d-5p, miR-1180-3p, miR-195-5p, and miR-223-3p) in the TCGA dataset (FDR ≤ 0.2; *t*-test).

**Figure 2:**
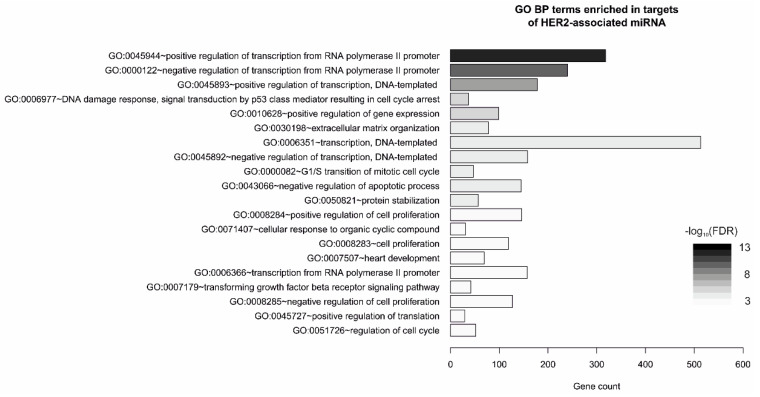
Gene Ontology Biological Process (GO BP) terms enriched in genes targeted by HER2-associated miRNAs in single hormone receptor-positive breast tumors; top20 terms/pathways with the lowest *p*-value plotted as the number of associated genes (gene count) and ordered according to -log 10(FDR); analyzed with Functional Annotation Tool by DAVID Bioinformatics Resources 6.81.

Functional analysis of gene ontology revealed that the predicted gene targets (3925; Supplementary Table S3) of HER2-associated miRNAs are mostly involved in transcription regulation, but also in cellular matrix organization, regulation of cell cycle or apoptosis; apart from cancer-associated pathways, the most altered signaling pathways included PI3-K-Akt, p53, and FoxO (Figure 2, Supplementary Table S4).

### 4.3. miRNAs Associated with Steroid Hormone Receptor Expression

In the NanoString cohort we did not identify any miRNA significantly different between both single hormone receptor-positive subgroups of breast cancer. Nevertheless, we observed a trend for differing expression (*p*-value ≤ 0.05; FDR > 0.2; ANOVA) in eight miRNAs. ER(+)/PgR(−) group demonstrated elevated levels of four miRNAs—miR-30a-5p, miR-29c-3p, miR-141-3p, and miR-423-5p—while the ER(−)/PgR(+) tumors were enriched in another four miRNAs—miR-514b-5p, miR-424-5p, miR-495-3p, miR-92a-3p (Table 4, Supplementary Figure S5). For one of the miRNAs—miR-29c-3p—the association with the ER(+)/PgR(−) phenotype was confirmed in the TCGA cohort (*p*-value = 0.024; *t*-test). Volcano plot of ER/PgR-associated miRNAs in single hormone receptor-positive breast tumors is shown in Supplementary Figure S6.

**Figure 3:**
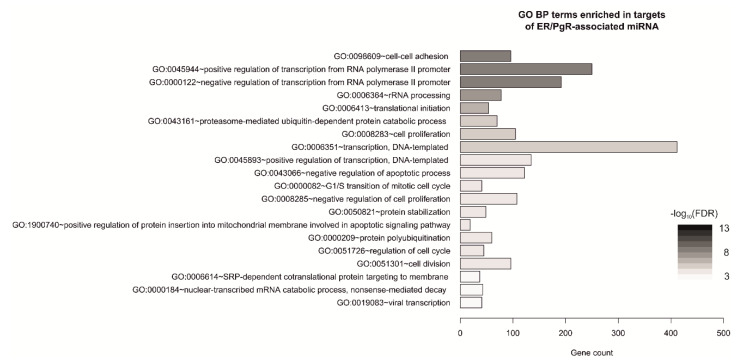
Gene Ontology Biological Process (GO BP) terms enriched in genes targeted by ER/PgR-associated miRNAs in single hormone receptor-positive breast cancers; top20 terms/pathways with the lowest *p*-value plotted as the number of associated genes (gene count) and ordered according to -log 10(FDR); analyzed with Functional Annotation Tool by DAVID Bioinformatics Resources 6.81.

Gene targets of the miRNAs potentially associated with the single hormone receptor-positive phenotype (3011; Supplemantary Table S3), were subjected to enrichment analysis. The identified pathways were mostly involved in cell–cell adhesion, as well as regulation of transcription, cell cycle and cell division (Figure 3, Supplementary Table S5). To further explore the association between selected miRNAs, their targets and enriched pathways, mRNA targets of ER/PgR-associated miRNAs (5/8 available in TCGA dataset) were matched with genes associated with a given GO BP term and the expression of each miRNA was correlated with expression of its mRNA targets. For each GO BP term, the overlap between mRNA correlating with miRNA of interest was illustrated with a Venn diagram (Supplementary Figure S7). The most significant effect (the number of selected miRNA targets involved in each pathway) was observed for miR-29c-3p and miR-141-3p.

Additionally, we analyzed mRNA-differentiating ER/PgR phenotypes based on the TCGA dataset, and found 10 genes uniquely differentiating between two subtypes of single hormone receptor-positive breast cancer (Supplementary Figure S8). Correlation between expression of ER/PgR-associated miRNA and targeted mRNA was estimated for four available miRNA-mRNA (*TGFB2*–miR-141-3p, *NEDD4L*–miR-30a-5p, *FGFR4*–miR-424-5p, *SOCS2*–miR-424-5p) was assessed, but no significant results were obtained (Supplementary Figure S9).

## 5. Discussion

In this pilot study of miRNA expression profiling in single hormone receptor-positive breast cancer we demonstrated that miRNA expression profiles of these tumors depend mainly on their HER2 status, rather than on their hormonal receptor status. However, we also found several candidate miRNAs which could be potentially associated with either an ER(−)/PgR(+) or an ER(+)/PgR(−) subtype of breast cancer, which may indicate their biological importance in these tumors.

Four miRNAs in our study showed a decreased expression in HER2-overexpressing/amplified tumors in both NanoString and TCGA cohorts. Three of them (miR-30d-5p, miR-195-5p, and miR-223-3p) were previously reported to be downregulated in HER2-overexpressing/amplified cancers [22,23,24]. miR-223-3p is also downregulated in HER2-overexpressing C5.2 cell line [25]. Citron et al. postulated a central role for miR-223 in the control of epidermal growth factor signaling and HER2 activation, as it reduces the oncogenic potential of HER2-transformed mammary epithelial cells [26]. Moreover, activation of HER2 downregulates miR-223-3p via RB repression and E2F1 activation [26]. Another miRNA—miR-30d—was upregulated by trastuzumab in BT474 cells [27]. In ovarian carcinoma, a lower expression of the miR-30 family, including miR-30d, was associated with HER2 overexpression [23]. Two miRNAs identified in our cohort—miR-30d and mir-195-5p—inhibit the cell cycle by targeting cyclin E [28,29]. Consistently, their lower expression was noted in biologically aggressive types of breast cancer, i.e., HER2-enriched and basal-like carcinomas [30]. As expected, G1/S transition of mitotic cycle and regulation of cell cycle were identified in the top 20 GO BP categories predicted as miRNA targets in our study. miR-1180-3p—the fourth marker validated in TCGA dataset—has not been observed to be associated with HER2 status to date.

Interactions between miRNAs and ER in breast cancer are mutually interrelated. Estrogen receptor interferes with the miRNA processing pathway by targeting Drosha complex, Argonaut proteins, and Dicer [10]. On the other hand, multiple miRNAs regulate the activity and expression of ER in breast cancer, which may translate into responsiveness to hormonal treatment. In our cohort, we observed upregulation of miR-92a-3p, a member of the miR-17-92 cluster, in the ER(−)/PgR(+) group. Its associations with ERα are unclear, but it directly downregulates ERβ in breast cancer [31]. In the Norwegian Women and Cancer study, miR-92a-3p was upregulated in triple-negative carcinomas [32]. So far, few studies have investigated interactions between PgR and miRNAs. One of the mechanisms of ER-dependent upregulation of PgR involves downregulation of miR-26a and miR-181a, which bind to *PGR* 3′UTR and repress its expression [33]. In line with this, Gilam et al. proposed that miR-181a, miR-23a, and miR-26b might be responsible for PgR downregulation in ER(+)/PgR(−) tumors [34], but this was not confirmed by our data. One preliminary study suggested miR-495 as a novel negative regulator of ER and PgR [35]. As the vast majority of ER(−)/PgR(+) breast cancer express low levels of PgR, it may suggest that in some cases miR-495-3p contributes to a lack of ER expression with retained low PgR expression.

ER(+)/PgR(−) tumors from our cohort demonstrated higher expression of miR-29c-3p, a member of an miRNA cluster recently connected with ER(+) luminal tumors, consisting also of miR-149, miR-375, and miR-26b [11]. Similarly, the levels of miR-30 family members positively correlate with ER and a lack of EGFR [29]. High expression of miR-30a is associated with a favorable response to tamoxifen and a longer progression-free survival [29]. Another identified miRNA—miR-141-3p—belongs to another cluster characteristic for ER(+) tumors together with miR-451 and miR-486 [36]. Interestingly, miR-141-3p has reciprocal interactions with PgR. Progesterone downregulates miR-141-3p leading to derepression of signal transducer and activator of transcription 5A (Stat5a), and subsequently to expansion of stem-like breast cancer cells [37]. On the other hand, depletion of miR-141-3p increases PgR levels, even in breast cancer cell lines where its expression is ER-dependent [37]. This suggests that miR-141-3p downregulation may be a crucial event in the maintenance of PgR expression in ER(-) tumors. 

**Figure 4:**
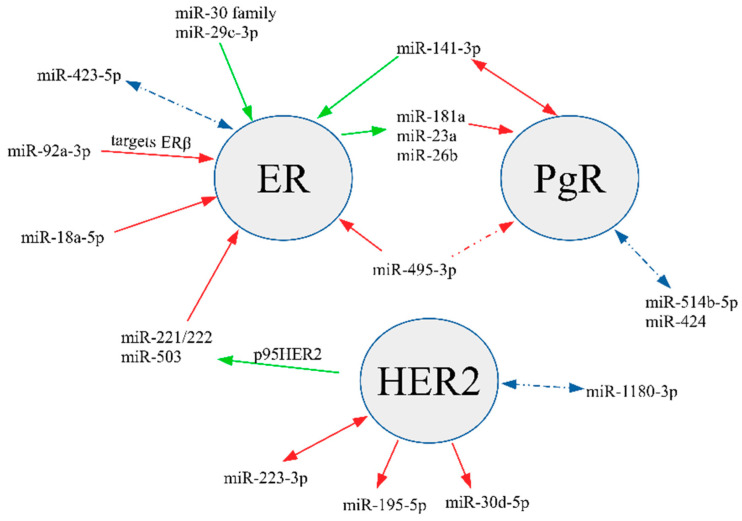
Key interactions between selected miRNAs, ER, PgR, and HER2. Red lines illustrate a known downregulating effect or an inverse association between miRNA and receptor; green lines depict an upregulating effect or a positive association between miRNA and receptor; blue lines show a potential relationship.

In 2009, Lowery et al. identified miRNA signatures predicting expression of ER, PgR, and HER2 in breast cancer [38]. They demonstrated an association between miR-520g, miR-377, miR-527-518a, and miR-520f-520c and PgR, whereas miR-342, miR-299, miR-217, miR-190, miR-135b, and miR-218 predicted ER expression. Our study investigated miR-342, miR-299, miR-135, miR-218, but we did not observe any significant differences in their levels between groups. Recent research indicates that some miRNAs may directly target and silence ER expression, e.g., miR-18a-5p and miR-222 [39], and thus may participate in the development of ER(−)/PgR(+) breast cancer. A recent study by Gorbatenko et al. demonstrated that p95HER2 induces miR-221/222 and miR-503, leading to decreased *ESR1* expression and enhanced invasion and migration [39]. Likewise, miR-18a-5p is upregulated in ER(−) tumors and decreases expression of ER by binding to its mRNA [40]. In the current study, we observed a trend of higher expression of both these miRNAs in the ER(−)/PgR(+) group, but these findings lacked statistical significance (data not shown). Other miRNAs identified as potentially upregulated in ER(−)/PgR(+) (miR-514b-5p and miR-424) and ER(+)/PgR(−) (miR-423-5p) have not been previously reported to show differential expression with regard to steroid hormones receptor profiles. The main interactions between the discussed miRNAs, ER, PgR, and HER2 are summarized in Figure 4.

Limitations:

This was a retrospective study enrolling a small, clinically heterogenous cohort determined by frequency of ER(−)/PgR(+) cancers in the population (~1% of all breast cancers). Moreover, our observations need further validation, as we were able to externally validate expression only of miRNAs overlapping between our study and TCGA data.

## 6. Conclusions

ER(−)/PgR(+) tumors show a profile resembling triple- and double-negative tumors, which may indicate that their biology is similar to basal-like carcinomas. On the contrary, ER(+)/PgR(−) tumors show a higher expression of miRNAs typical for double-positive luminal carcinomas. The main differences in miRNA expression amongst single hormone receptor-positive tumors were, however, related to their HER2 status. Further multicenter studies are necessary to comprehensively analyze miRNA signatures characteristic for ER(−)/PgR(+) and ER(+)/PgR(−) tumors.

## Figures and Tables

**Table 1:** Clinicopathological characteristics of patients with single hormone receptor-positive breast tumors in the NanoString cohort; differences estimated with *t*-test (age, Ki67, tumor size) or Fisher’s Exact test (grade, estrogen receptor (ER) status, progesterone receptor (PgR) status, human epidermal growth factor receptor 2 (HER2) status, T, N, M); significant results (*p*-value < 0.05) are in bold.

Parameter		All (*n* = 32)		ER(+)PgR(−) (*n* = 14)		ER(−)PgR(+) (*n* = 18)		*p*-Value
Age	median (range)	62	29–78	66	36–76	53.5	29–78	0.141
Grade	1	0	0%	0	0%	0	0%	**0.003**
	2	6	19%	6	19%	0	0%	
	3	26	81%	8	25%	18	56%	
ER status	negative	18	56%	0	0%	18	56%	**<0.001**
	positive	14	44%	14	44%	0	0%	
PgR status	negative	14	44%	14	44%	0	0%	**<0.001**
	positive	18	56%	0	0%	18	56%	
HER2 status	negative	15	47%	5	16%	10	31%	0.308
	positive	17	53%	9	28%	8	25%	
Ki67	median (range)	47.5	9–90	30	9–70	60	30–90	**<0.001**
Tumor size [mm]	median (range)	21	8–47	21.5	12–30	21	8–47	0.216
T	1	14	44%	6	19%	8	25%	0.963
	2	13	41%	6	19%	7	22%	
	3	1	3%	1	3%	0	0%	
	4	3	9%	1	3%	2	6%	
	NA	1	3%	0	0%	1	3%	
N	0	17	53%	8	25%	9	28%	0.351
	1	11	34%	6	19%	5	16%	
	2	3	9%	0	0%	3	9%	
	NA	1	3%	0	0%	1	3%	
M	0	29	91%	13	41%	16	50%	1.000
	1	2	6%	1	3%	1	3%	
	NA	1	3%	0	0%	1	3%	

**Table 2:** Clinicopathological characteristics of patients with single hormone receptor-positive breast tumors in a TCGA breast invasive carcinoma cohort; differences estimated with *t*-test (age, Ki67, tumor size) or Fisher’s Exact test (ER status, PgR status, HER2 status, T, N, M); significant results (*p*-value < 0.05) are in bold.

Parameter		All (*n* = 67)		ER(+)PgR(−) (*n* = 57)		ER(−)PgR(+) (*n* = 10)		*p*-Value
Age	median (range)	60	30–90	61	30–90	55.5	46–90	0.486
ER status	negative	10	15%	0	0%	10	15%	**<0.001**
	positive	57	85%	57	85%	0	0%	
PgR status	negative	57	85%	57	85%	0	0%	**<0.001**
	positive	10	15%	0	0%	10	15%	
HER2 status	negative	53	79%	46	69%	7	10%	0.425
	positive	14	21%	11	16%	3	4%	
T	1	13	19%	12	18%	1	1%	0.437
	2	44	66%	35	52%	9	13%	
	3	9	13%	9	13%	0	0%	
	4	1	1%	1	1%	0	0%	
	NA	0	0%	0	0%	0	0%	
N	0	35	52%	25	37%	10	15%	**0.028**
	1	22	33%	22	33%	0	0%	
	2	3	4%	3	4%	0	0%	
	3	5	7%	5	7%	0	0%	
	NA	2	3%	2	3%	0	0%	
M	0	48	72%	39	58%	9	13%	0.260
	1	0	0%	0	0%	0	0%	
	NA	19	28%	18	27%	1	1%	

**Table 3:** HER2-associated miRNAs in single hormone receptor-positive breast tumors; log2fold change (log2FC) calculated for the mean normalized counts of each probe in compared groups—HER2(+) vs. HER2(−); miRNAs upregulated in HER2(+) tumors are marked with ↑, miRNAs downregulated in HER2(+) tumors are marked with ↓; differences estimated with Two-Way ANOVA (*p*-value) with Benjamini–Hochberg correction (FDR), only statistically significant results (FDR ≤ 0.2) are presented; miRNA names are according to miRBase database (v21).

miRNA	HER2 log2FC	Direction	HER2 *p*-Value	HER2 FDR	ER/PgR log2FC	ER/PgR *p*-Value	ER/PgR FDR
hsa-miR-887-5p	2.85	↑	0.003	0.130	0.42	0.990	0.998
hsa-miR-208a-3p	2.50	↑	0.012	0.156	0.79	0.541	0.847
hsa-miR-891a-5p	2.29	↑	0.020	0.160	0.85	0.473	0.826
hsa-miR-301a-5p	2.17	↑	0.026	0.160	1.26	0.178	0.811
hsa-miR-33b-5p	2.09	↑	0.024	0.160	0.69	0.598	0.864
hsa-miR-1296-3p	1.91	↑	0.007	0.130	0.53	0.675	0.898
hsa-miR-378d	1.91	↑	0.002	0.130	0.14	0.702	0.908
hsa-miR-548v	1.82	↑	0.027	0.160	0.82	0.400	0.811
hsa-miR-1295a	1.73	↑	0.005	0.130	0.73	0.338	0.811
hsa-miR-337-3p	1.54	↑	0.014	0.156	−0.11	0.480	0.827
hsa-miR-514b-5p	0.83	↑	0.023	0.160	−0.67	0.046	0.809
hsa-miR-185-5p	−0.71	↓	0.032	0.186	0.13	0.410	0.811
hsa-miR-340-5p	−0.72	↓	0.025	0.160	−0.27	0.666	0.898
hsa-miR-424-5p	−0.74	↓	0.020	0.160	−0.69	0.048	0.809
hsa-miR-106a-5p+hsa-miR-17-5p	−0.82	↓	0.007	0.130	−0.40	0.379	0.811
hsa-miR-151a-3p	−0.84	↓	0.021	0.160	0.29	0.221	0.811
hsa-miR-374a-5p	−0.91	↓	0.013	0.156	0.10	0.435	0.811
hsa-miR-141-3p	−0.92	↓	0.034	0.193	1.10	0.018	0.809
hsa-miR-26b-5p	−0.96	↓	0.025	0.160	0.54	0.110	0.809
hsa-miR-126-3p	−0.97	↓	0.006	0.130	0.20	0.255	0.811
hsa-miR-32-5p	−1.04	↓	0.017	0.160	0.19	0.349	0.811
hsa-miR-15b-5p	−1.08	↓	0.023	0.160	−0.55	0.425	0.811
hsa-miR-30d-5p	−1.12	↓	0.006	0.130	0.53	0.078	0.809
hsa-miR-1180-3p	−1.18	↓	0.008	0.142	−0.16	0.867	0.953
hsa-miR-30b-5p	−1.25	↓	0.002	0.130	0.13	0.310	0.811
hsa-miR-195-5p	−1.33	↓	0.024	0.160	0.49	0.209	0.811
hsa-miR-429	−1.39	↓	0.023	0.160	−0.27	0.997	0.998
hsa-miR-503-5p	−1.46	↓	0.011	0.156	−1.19	0.079	0.809
hsa-miR-223-3p	−1.47	↓	0.025	0.160	−1.30	0.087	0.809
hsa-miR-450a-5p	−1.60	↓	0.006	0.130	−0.74	0.397	0.811
hsa-miR-29b-3p	−1.63	↓	0.001	0.130	0.36	0.135	0.811
hsa-miR-135b-5p	−2.17	↓	0.016	0.160	−1.46	0.184	0.811
hsa-miR-660-5p	−2.30	↓	0.010	0.147	−1.49	0.167	0.811

**Table 4:** ER/PgR-associated miRNAs in single hormone receptor-positive breast cancers; log2fold change (log2FC) calculated for the mean normalized counts of each probe in compared groups—ER(+)/PgR(−) vs. ER(−)/PgR(+); miRNAs upregulated in ER(+)/PgR(−) tumors are marked with ↑, miRNAs downregulated in ER(+)/PgR(−) tumors are marked with ↓; differences estimated with Two-Way ANOVA (*p*-value) with Benjamini–Hochberg correction (FDR), only statistically significant results (*p*-value ≤ 0.05) are presented; miRNA names according to the miRBase database (v21).

miRNA	ER/PgR log2FC	Direction	ER/PgR *p*-Value	ER/PgR FDR	HER2 log2FC	HER2 *p*-Value	HER2 FDR
hsa-miR-30a-5p	1.91	↑	0.031	0.809	−1.69	0.046	0.221
hsa-miR-29c-3p	1.40	↑	0.030	0.809	−1.23	0.047	0.221
hsa-miR-141-3p	1.10	↑	0.018	0.809	−0.92	0.034	0.193
hsa-miR-423-5p	0.73	↑	0.045	0.809	−0.49	0.119	0.338
hsa-miR-514b-5p	−0.67	↓	0.046	0.809	0.83	0.023	0.160
hsa-miR-424-5p	−0.69	↓	0.048	0.809	−0.74	0.020	0.160
hsa-miR-495-3p	−2.05	↓	0.027	0.809	−0.51	0.773	0.851
hsa-miR-92a-3p	−2.32	↓	0.033	0.809	−1.27	0.206	0.419

## Supplementary Materials

The following are available online at https://www.mdpi.com/2075-4418/10/9/617/s1, **Supplementary Table S1.** List of patients (barcode IDs) analyzed for miRNA and mRNA expression in TCGA dataset along with ER/PgR/HER2 characterization of primary tumor; **Supplementary Table S2.** List of miRNA analyzed in NanoString and TCGA datasets (*n* = 185), including: HER2- (*n* = 33) and ER/PgR-associated (*n* = 8) miRNA in NanoString dataset; HER2- (*n* = 23) and ER/PgR-associated (*n* = 5) miRNA available for validation in TCGA dataset; miRNA names according to miRBase database v21 (NanoString) and v16 (TCGA); **Supplementary Table S3.** List of gene targets of HER2- (*n* = 33) and ER/PgR-associated (*n* = 8) miRNA in mammary gland tissue generated using miRNET 2.0 database; **Supplementary Table S4.** List of GO BP enriched in genes targeted by HER2-associated miRNAs in single hormone receptor positive breast tumors generated using Functional Annotation Tool by DAVID Bioinformatics Resources 6.81; **Supplementary Table S5.** List of GO BP enriched in genes targeted by ER/PgR-associated miRNAs in single hormone receptor positive breast tumors generated using Functional Annotation Tool by DAVID Bioinformatics Resources 6.81; **Supplementary Figure S1.** The chart presenting the number of miRNAs included in the study; **Supplementary Figure S2.** Distribution of clinicopathological characteristics of patients with single hormone receptor positive breast tumors in NanoString (*n* = 32) and TCGA cohorts (*n* = 67); differences estimated with *t*-test (age) or Fisher’s Exact test (HER2 status, T, N, M); **Supplementary Figure S3.** Expression of HER2-associated miRNAs according to the ER/PgR/HER2 status (E—ER; P—PgR); expression depicted as number of counts of each probe normalized to all miRNA genes; differences estimated with Two-Way ANOVA (*p*-value) with Benjamini–Hochberg correction (FDR); bars correspond to IQR, whiskers cover 1.5 IQR from the median; **Supplementary Figure S4.** Volcano plot of HER2-associated miRNAs in single hormone receptor-positive breast tumors in NanoString cohort; for each miRNA -log10(*p*-value) plotted against log2FC; miRNAs upregulated in HER2(+) tumors marked in red, miRNAs downregulated in HER2(-) tumors marked in blue; *p*-value cut-off (-log10(*p*-value) = 1.45; matching FDR = 0.19) represented by a grey horizontal line; up/downregulation cut-offs (log2FC > 0.3 and log2FC < −0.3, respectively) represented by grey vertical lines; **Supplementary Figure S5.** Expression of ER/PgR-associated miRNAs according to the ER/PgR/HER2 status (E—ER; P—PgR); expression depicted as number of counts of each probe normalized to all miRNA genes; differences estimated with Two-Way ANOVA (*p*-value) with Benjamini–Hochberg correction (FDR); bars correspond to IQR, whiskers cover 1.5 IQR from the median; **Supplementary Figure S6.** Volcano plot of ER/PgR-associated miRNAs in single hormone receptor positive breast tumors in NanoString cohort; for each miRNA -log10(*p*-value) plotted against log2FC; miRNAs upregulated in ER(+)/PgR(−) tumors marked in red, miRNAs downregulated in ER(+)/PgR(−) tumors marked in blue; *p*-value cut-off represented by grey horizontal line; up/downregulation cut-offs (log2FC > 0.3 and log2FC < −0.3, respectively) represented by grey vertical lines; **Supplementary Figure S7.** Reciprocal expression of ER/PgR-associated miRNA and their mRNA targets in TCGA dataset; for each of top20 GO BP terms enriched in NanoString cohort, mRNA targets of ER/PgR-associated miRNAs (5/8 available in TCGA dataset) were matched with genes associated with given term and the expression of each miRNA was correlated with expression of its mRNA targets; for each GO BP term, the overlap between mRNA correlating with miRNA of interest (cor > 0.3 or cor < −0.3) was illustrated with a Venn diagram; GO BP terms with at least 5 mRNA targets per each miRNA are presented (12/20, A-L); **Supplementary Figure S8.** mRNA differentiating ER/PgR phenotypes of breast tumors in TCGA dataset; transcriptome profiles of four ER/PgR phenotypes were compared and a list of differentiating genes (log2FC > 1 or log2FC < −1; FDR < 0.05, *t*-test) was generated for each comparison; the overlap between mRNA differentiating each pair of ER/PgR phenotypes was illustrated with a Venn diagram; **Supplementary Figure S9.** Correlation between expression of ER/PgR-associated miRNA and targeted mRNA that were uniquely differential for ER(+)/PgR(−) from ER(−)/PgR(+) breast tumors in TCGA dataset; miRNA-mRNA correlation was estimated for four available miRNA-mRNA pairs using Pearson’s method; ER(+)/PR(−) tumors are marked in red; ER(−)/PR(+) tumors are marked in blue.
