# Supplementary material for: microRNA Expression Profile in Single Hormone Receptor-Positive Breast Cancers Is Mainly Dependent on HER2 Status—A Pilot Study
Source: Diagnostics (Basel). 2020 Aug 20;10(9):617. doi: 10.3390/diagnostics10090617 (PMC7555149; doi:10.3390/diagnostics10090617)
Supplement: Supplementary file 1 [file diagnostics-10-00617-s001.zip › Sup_Fig_3.pdf]

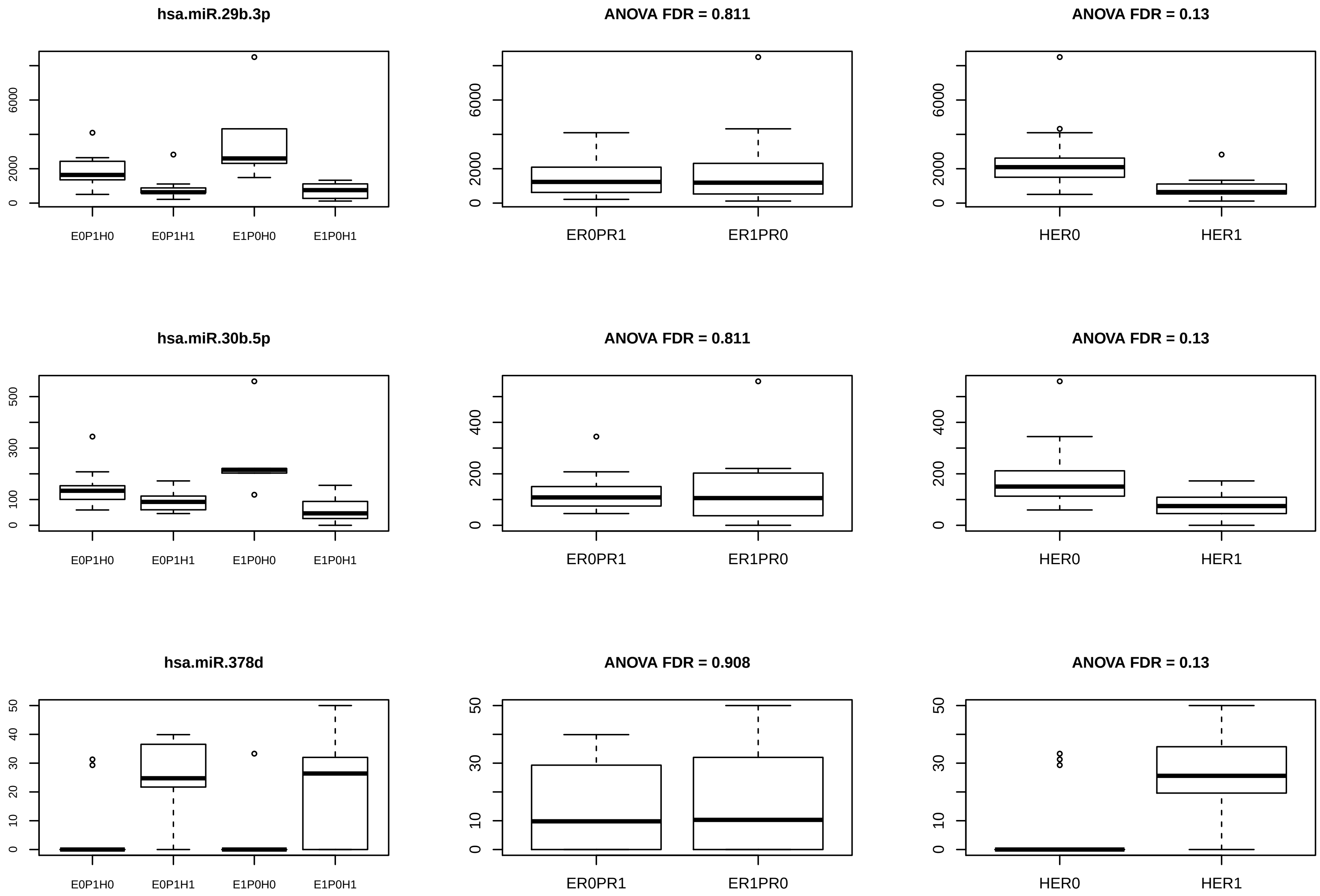

hsa.miR.29b.3p
ANOVA FDR = 0.811
ANOVA FDR = 0.13
E0P1H0
E0P1H1
E1P0H0
E1P0H1
ER0PR1
ER1PR0
HER0
HER1
hsa.miR.30b.5p
ANOVA FDR = 0.811
ANOVA FDR = 0.13
hsa.miR.378d
ANOVA FDR = 0.908
ANOVA FDR = 0.13

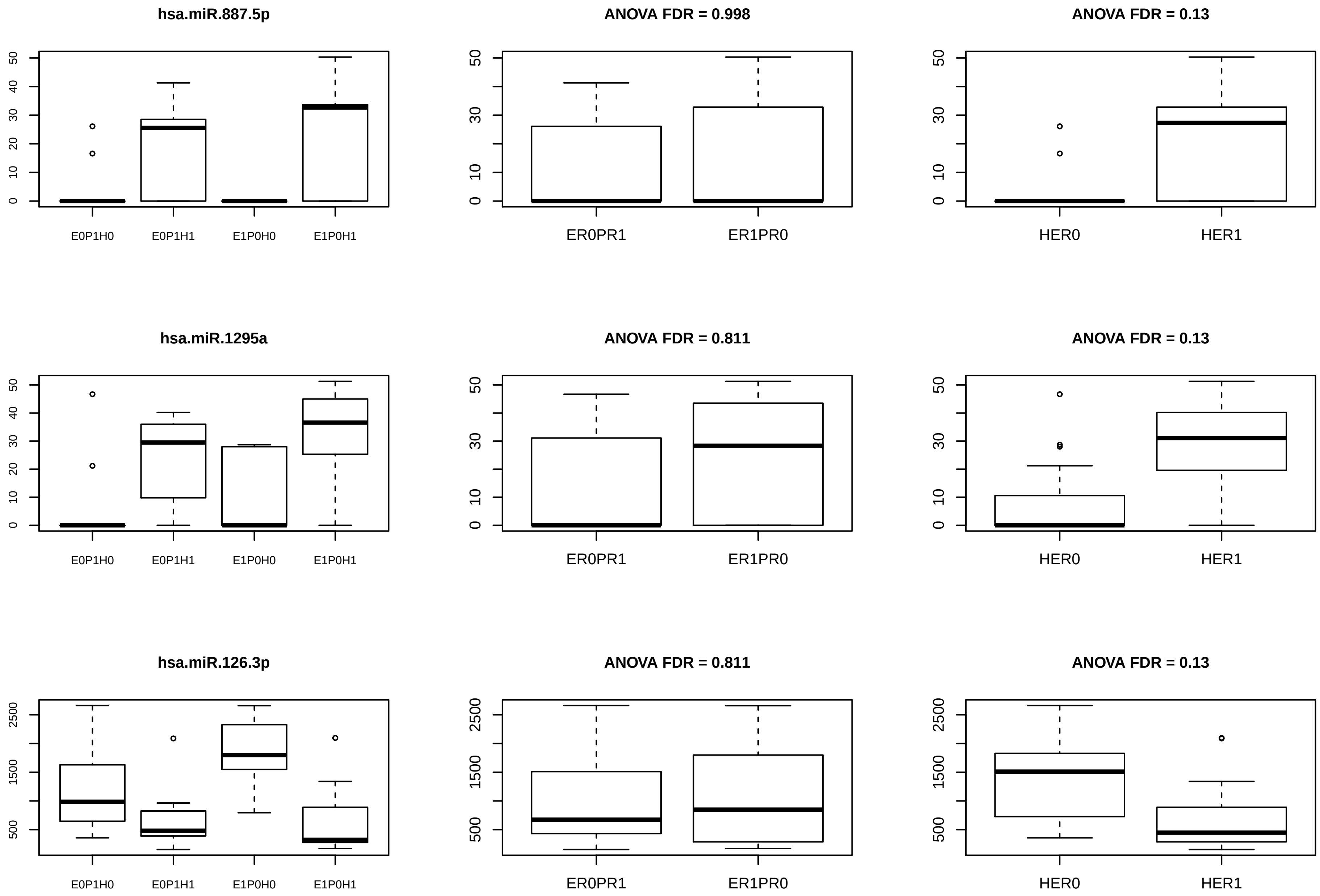
hsa.miR.887.5p
ANOVA FDR = 0.998
ANOVA FDR = 0.13
0
10
20
30
40
50
E0P1H0
E0P1H1
E1P0H0
E1P0H1
ER0PR1
ER1PR0
HER0
HER1
hsa.miR.1295a
ANOVA FDR = 0.811
ANOVA FDR = 0.13
hsa.miR.126.3p
ANOVA FDR = 0.811
ANOVA FDR = 0.13
500
1500
2500

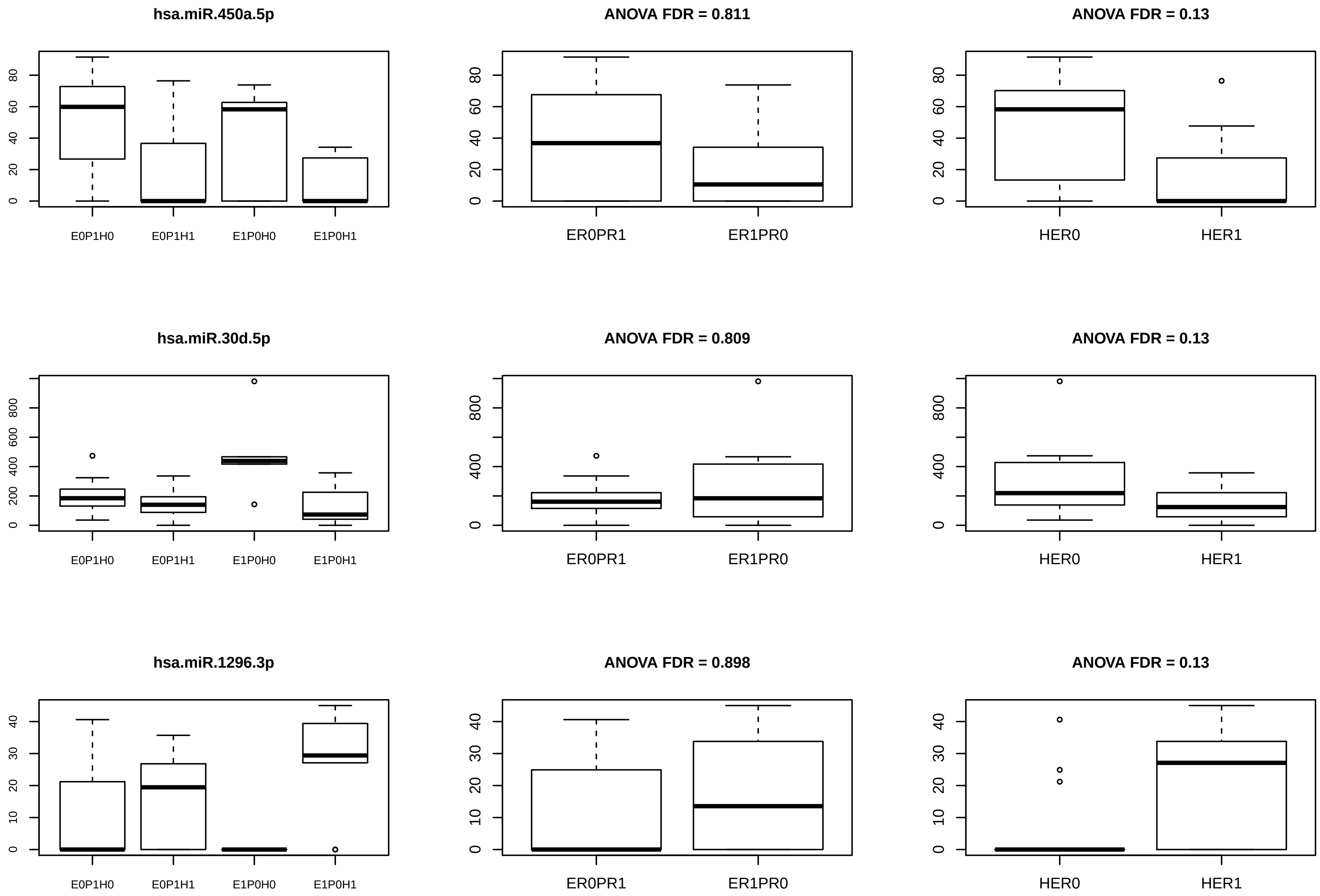
hsa.miR.450a.5p
ANOVA FDR = 0.811
ANOVA FDR = 0.13
E0P1H0
E0P1H1
E1P0H0
E1P0H1
ER0PR1
ER1PR0
HER0
HER1
hsa.miR.30d.5p
ANOVA FDR = 0.809
ANOVA FDR = 0.13
hsa.miR.1296.3p
ANOVA FDR = 0.898
ANOVA FDR = 0.13

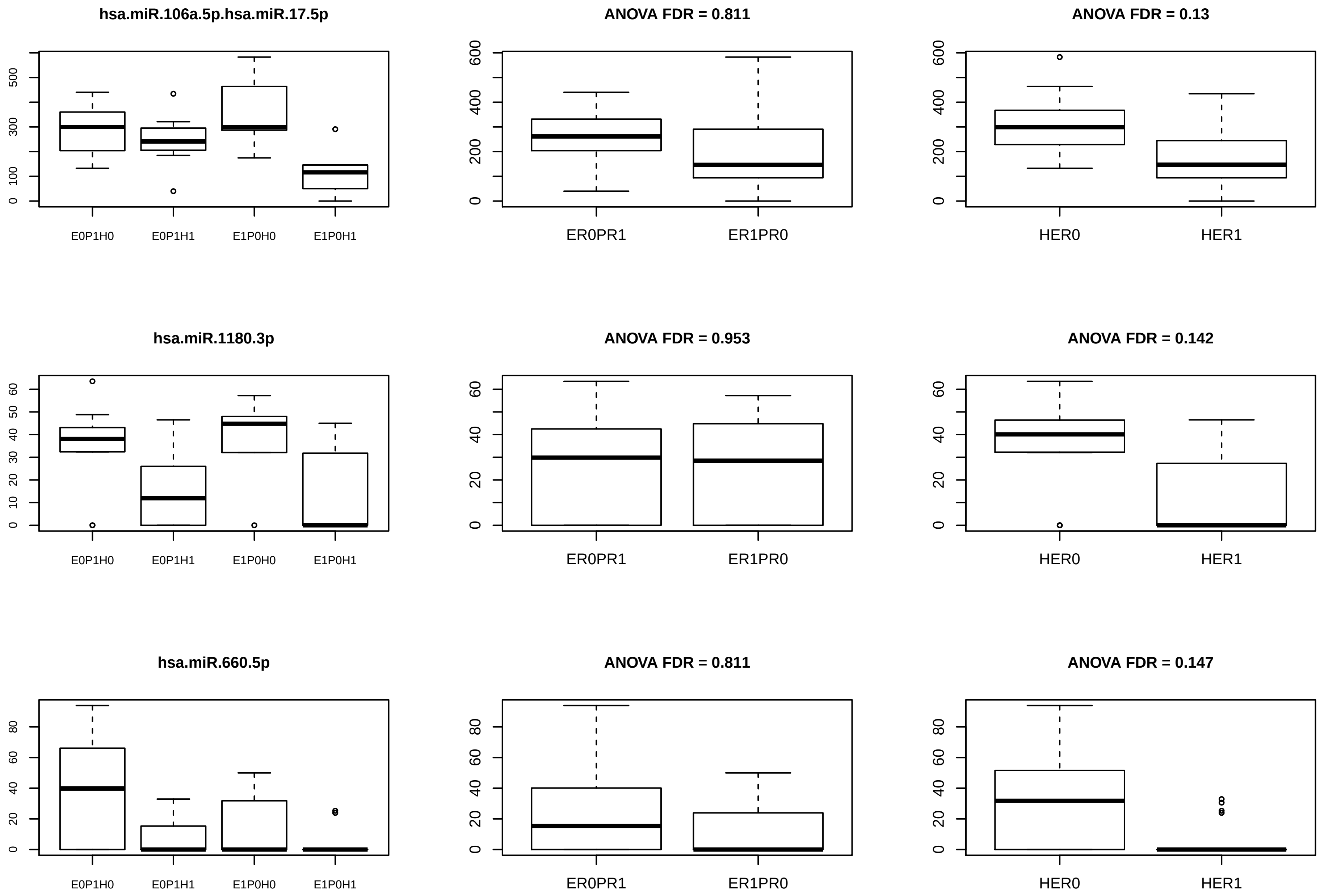
hsa.miR.106a.5p.hsa.miR.17.5p
ANOVA FDR = 0.811
ANOVA FDR = 0.13
E0P1H0
E0P1H1
E1P0H0
E1P0H1
ER0PR1
ER1PR0
HER0
HER1
hsa.miR.1180.3p
ANOVA FDR = 0.953
ANOVA FDR = 0.142
E0P1H0
E0P1H1
E1P0H0
E1P0H1
ER0PR1
ER1PR0
HER0
HER1
hsa.miR.660.5p
ANOVA FDR = 0.811
ANOVA FDR = 0.147
E0P1H0
E0P1H1
E1P0H0
E1P0H1
ER0PR1
ER1PR0
HER0
HER1

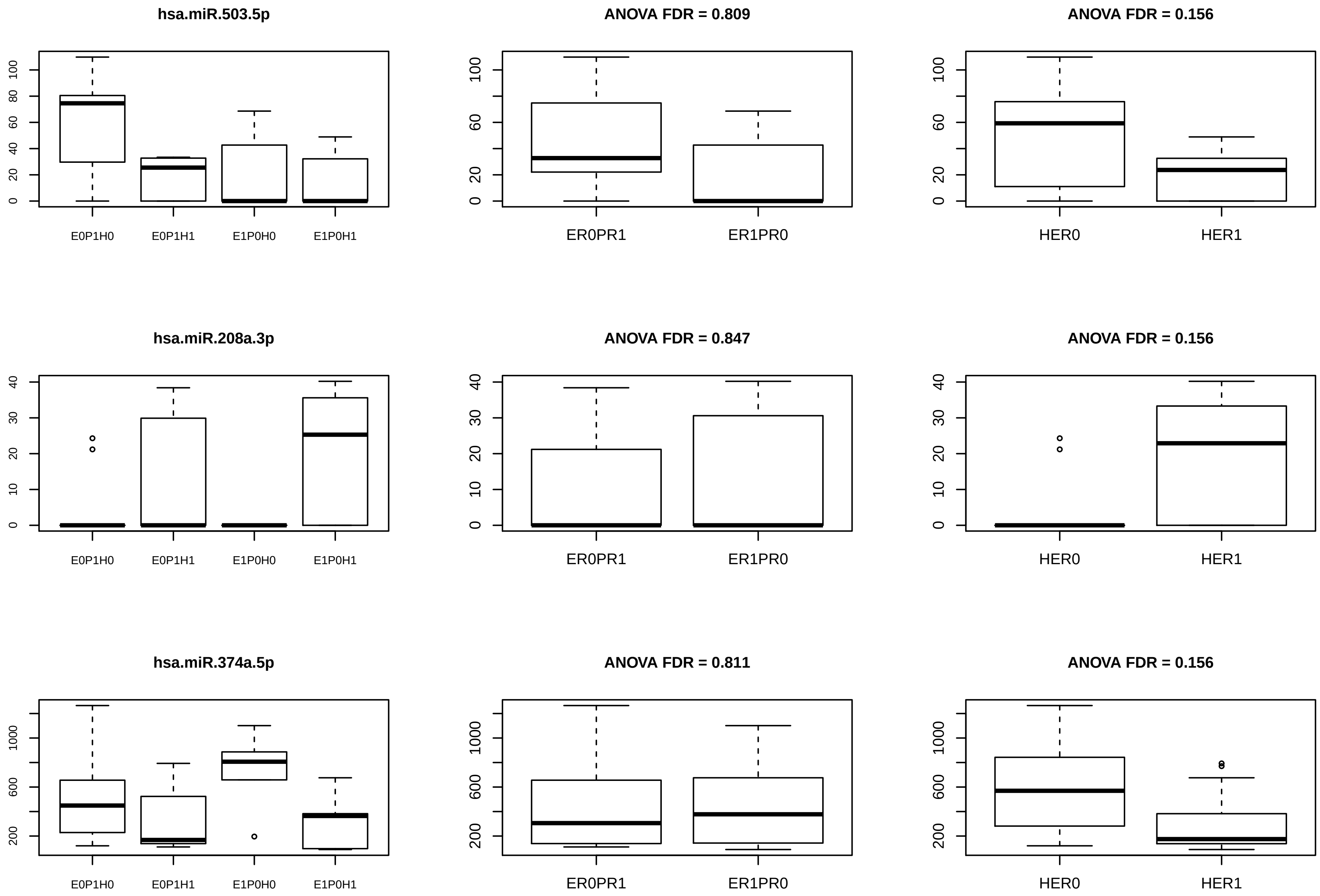
hsa.miR.503.5p
ANOVA FDR = 0.809
ANOVA FDR = 0.156
E0P1H0
E0P1H1
E1P0H0
E1P0H1
ER0PR1
ER1PR0
HER0
HER1
hsa.miR.208a.3p
ANOVA FDR = 0.847
ANOVA FDR = 0.156
E0P1H0
E0P1H1
E1P0H0
E1P0H1
ER0PR1
ER1PR0
HER0
HER1
hsa.miR.374a.5p
ANOVA FDR = 0.811
ANOVA FDR = 0.156
E0P1H0
E0P1H1
E1P0H0
E1P0H1
ER0PR1
ER1PR0
HER0
HER1

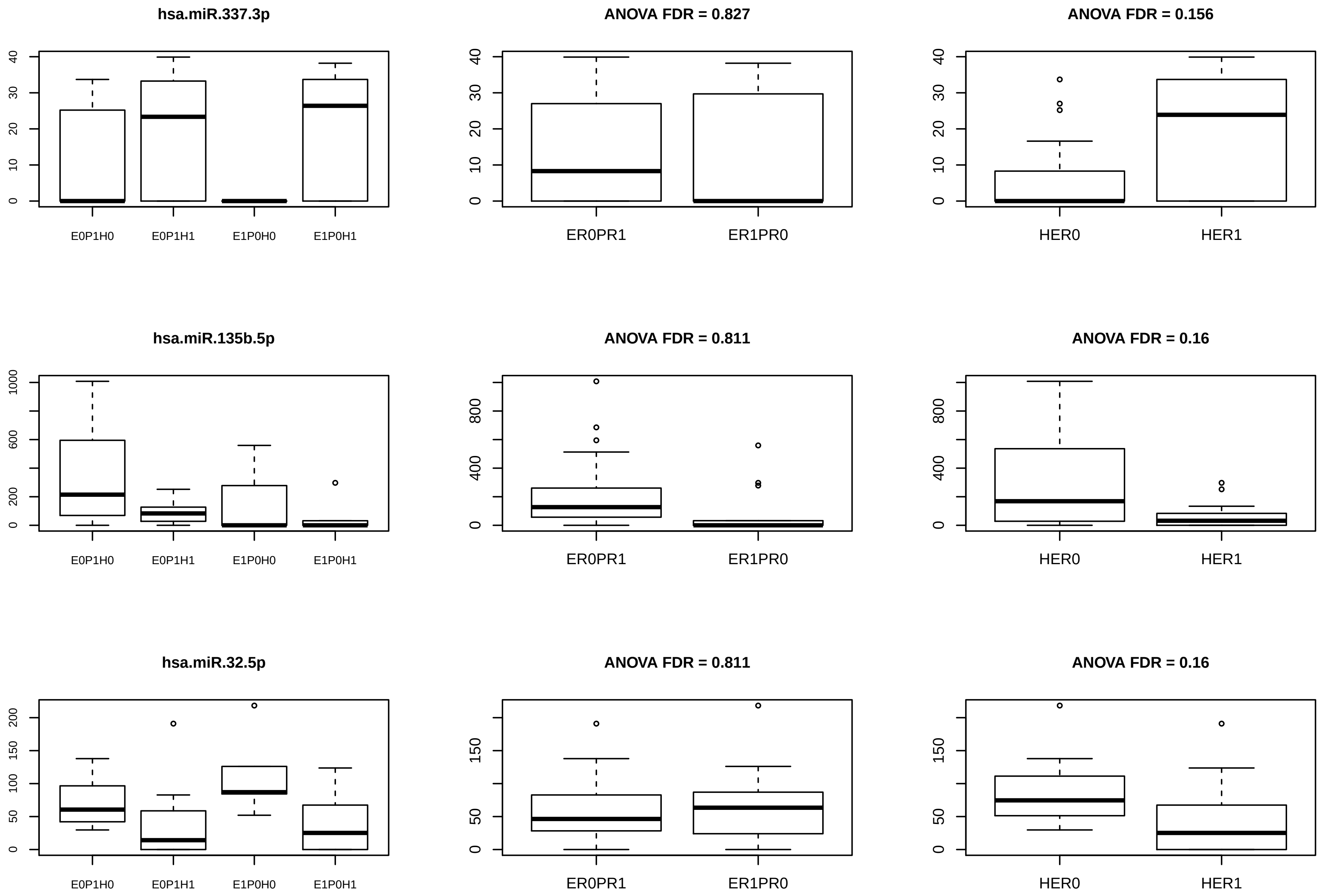
hsa.miR.337.3p
ANOVA FDR = 0.827
ANOVA FDR = 0.156
0
10
20
30
40
E0P1H0
E0P1H1
E1P0H0
E1P0H1
ER0PR1
ER1PR0
HER0
HER1
hsa.miR.135b.5p
ANOVA FDR = 0.811
ANOVA FDR = 0.16
0
200
400
600
800
1000
hsa.miR.32.5p
ANOVA FDR = 0.811
ANOVA FDR = 0.16
0
50
100
150
200

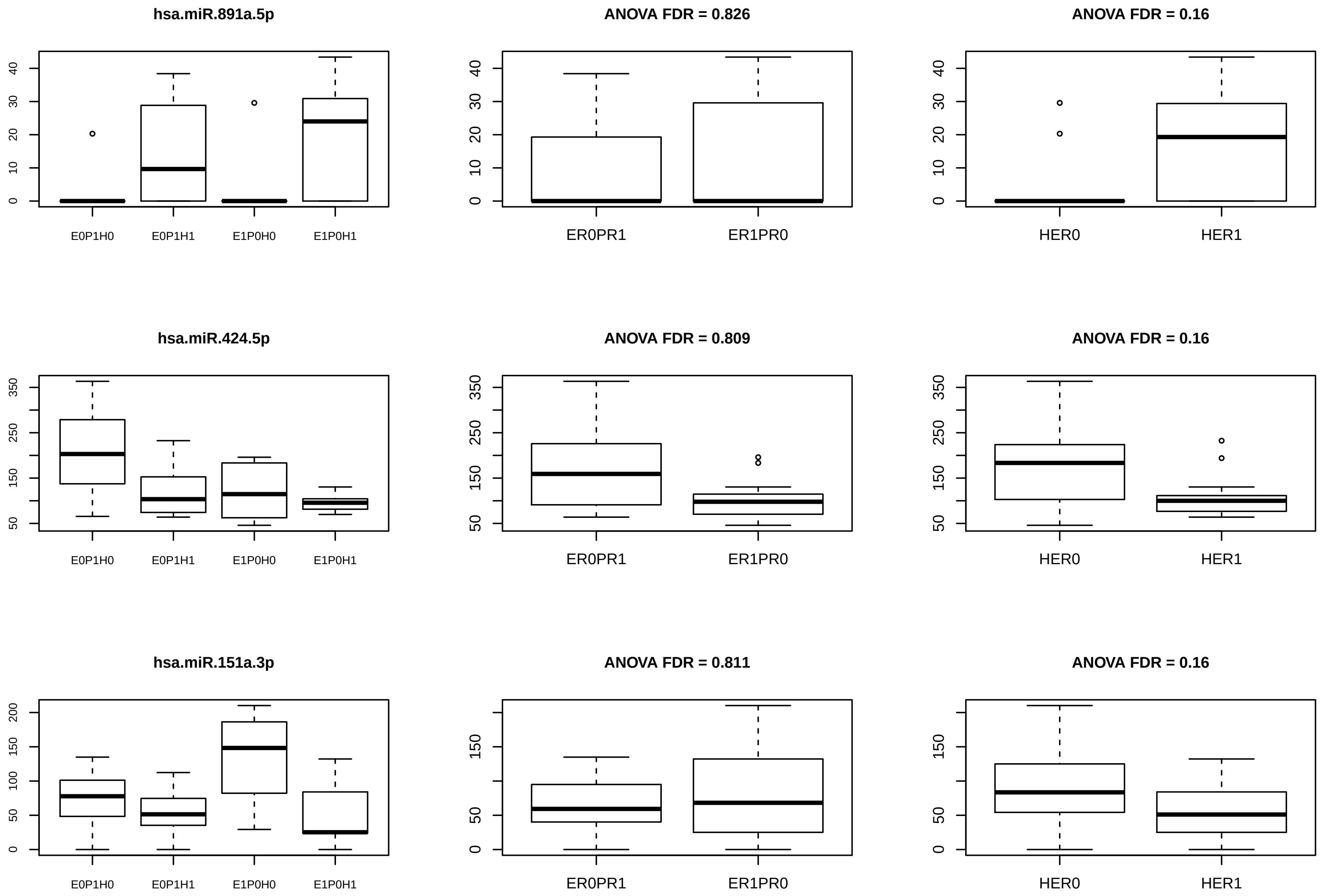
hsa.miR.891a.5p
ANOVA FDR = 0.826
ANOVA FDR = 0.16
E0P1H0
E0P1H1
E1P0H0
E1P0H1
ER0PR1
ER1PR0
HER0
HER1
hsa.miR.424.5p
ANOVA FDR = 0.809
ANOVA FDR = 0.16
E0P1H0
E0P1H1
E1P0H0
E1P0H1
ER0PR1
ER1PR0
HER0
HER1
hsa.miR.151a.3p
ANOVA FDR = 0.811
ANOVA FDR = 0.16
E0P1H0
E0P1H1
E1P0H0
E1P0H1
ER0PR1
ER1PR0
HER0
HER1

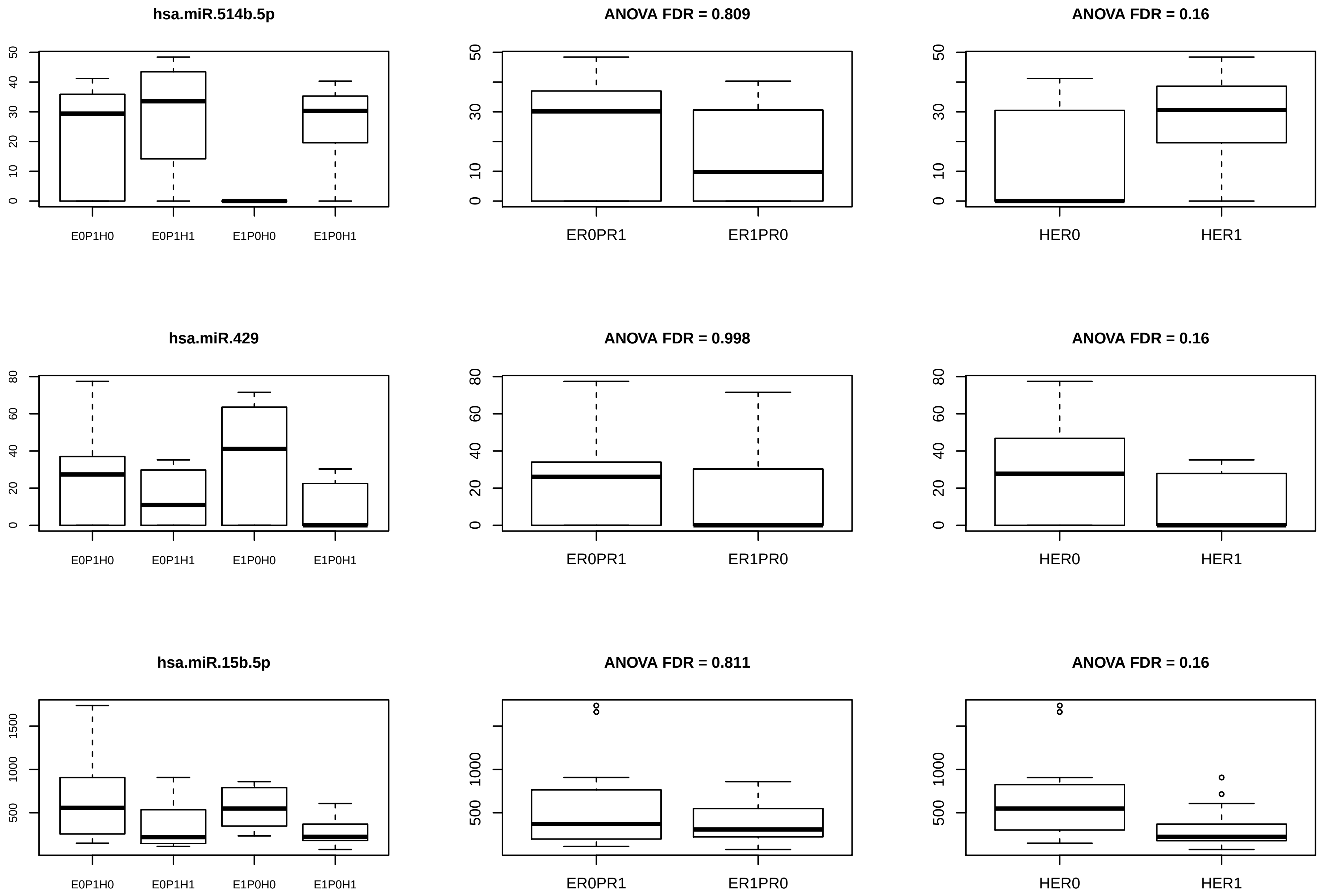
hsa.miR.514b.5p
ANOVA FDR = 0.809
ANOVA FDR = 0.16
E0P1H0
E0P1H1
E1P0H0
E1P0H1
ER0PR1
ER1PR0
HER0
HER1
hsa.miR.429
ANOVA FDR = 0.998
ANOVA FDR = 0.16
hsa.miR.15b.5p
ANOVA FDR = 0.811
ANOVA FDR = 0.16

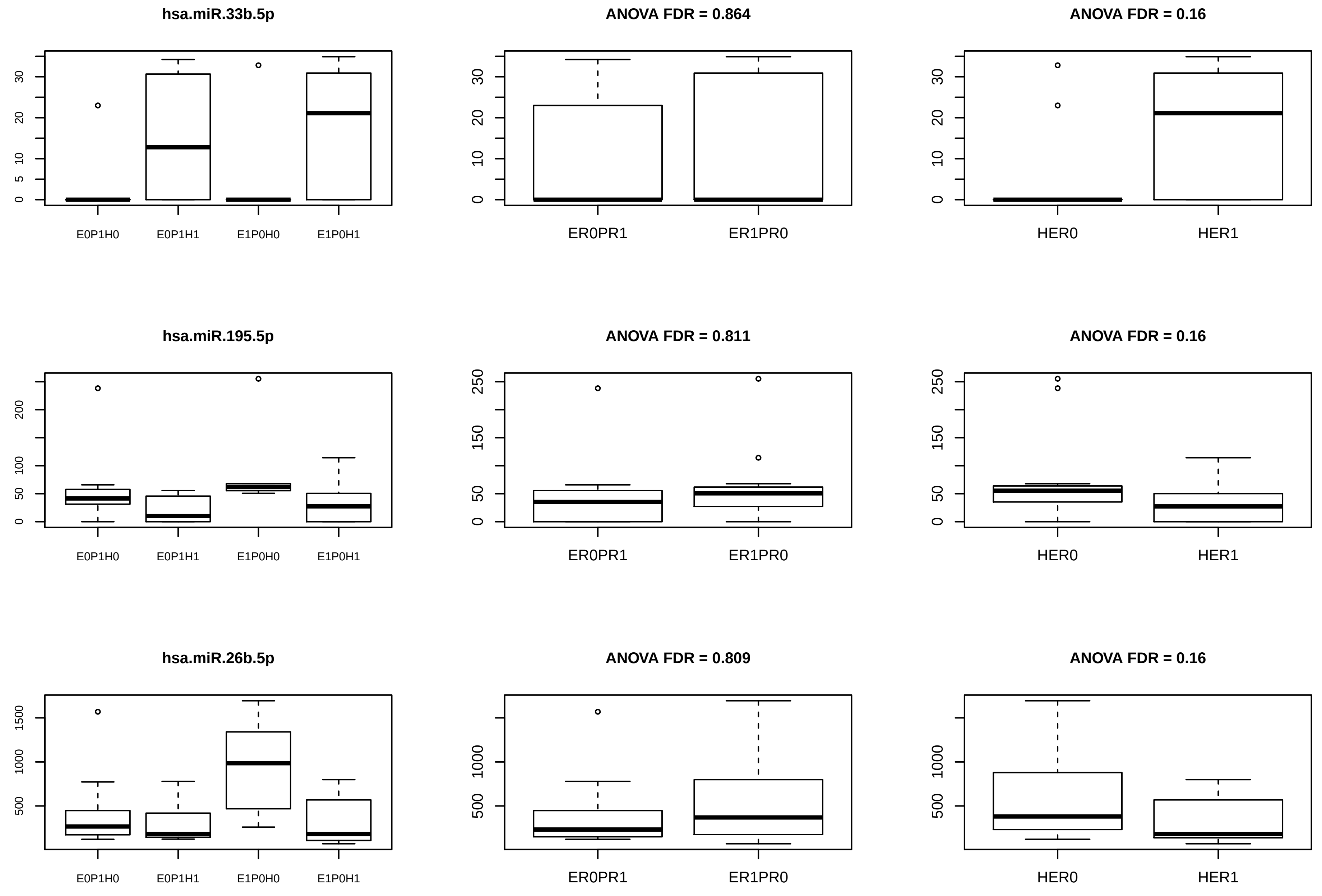
hsa.miR.33b.5p
0
5
10
20
30
E0P1H0
E0P1H1
E1P0H0
E1P0H1
ANOVA FDR = 0.864
0
10
20
30
ER0PR1
ER1PR0
ANOVA FDR = 0.16
0
10
20
30
HER0
HER1
hsa.miR.195.5p
0
50
100
200
E0P1H0
E0P1H1
E1P0H0
E1P0H1
ANOVA FDR = 0.811
0
50
150
250
ER0PR1
ER1PR0
ANOVA FDR = 0.16
0
50
150
250
HER0
HER1
hsa.miR.26b.5p
500
1000
1500
E0P1H0
E0P1H1
E1P0H0
E1P0H1
ANOVA FDR = 0.809
500
1000
ER0PR1
ER1PR0
ANOVA FDR = 0.16
500
1000
HER0
HER1

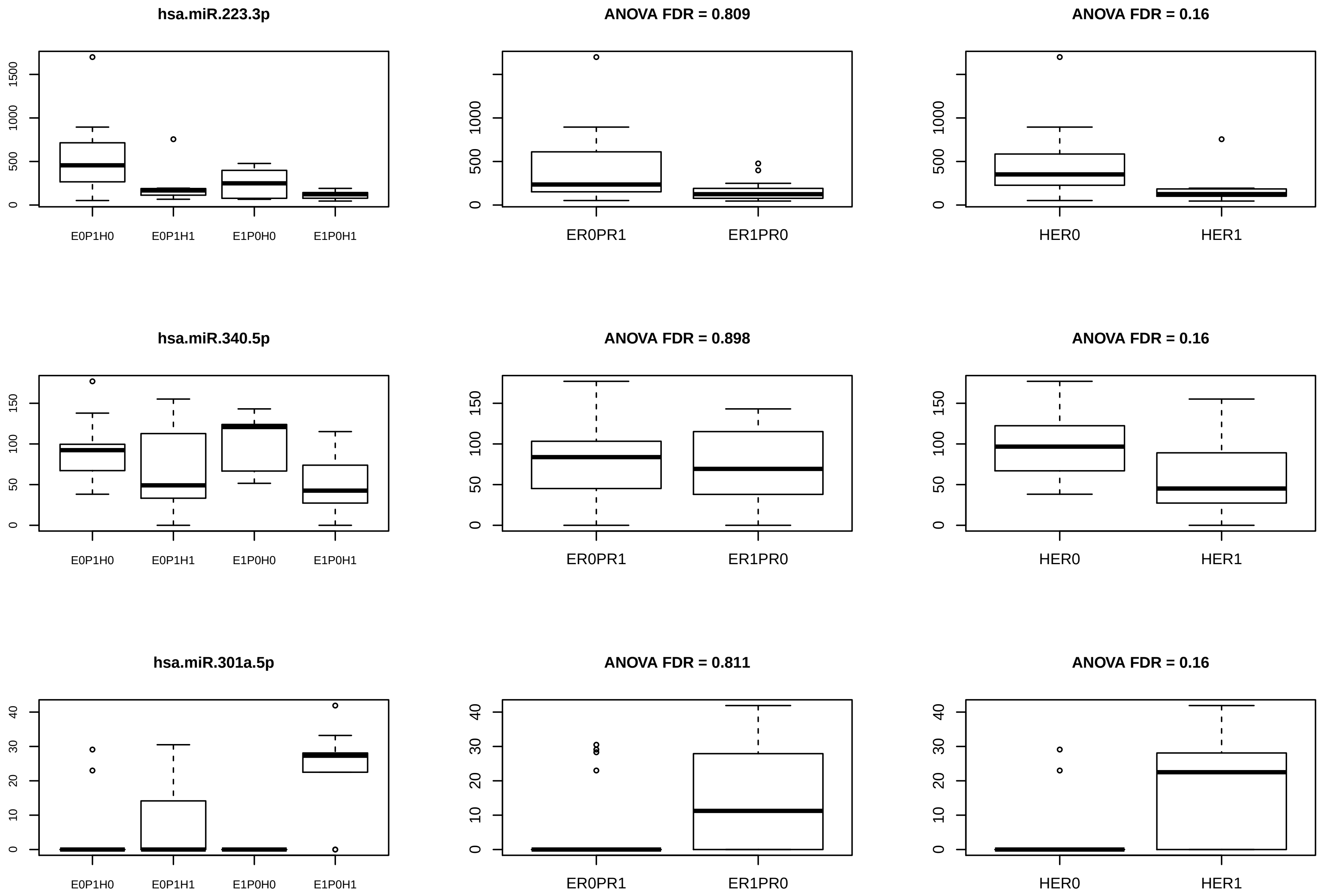
hsa.miR.223.3p
ANOVA FDR = 0.809
ANOVA FDR = 0.16
0
500
1000
1500
E0P1H0
E0P1H1
E1P0H0
E1P0H1
ER0PR1
ER1PR0
HER0
HER1
hsa.miR.340.5p
ANOVA FDR = 0.898
ANOVA FDR = 0.16
0
50
100
150
E0P1H0
E0P1H1
E1P0H0
E1P0H1
ER0PR1
ER1PR0
HER0
HER1
hsa.miR.301a.5p
ANOVA FDR = 0.811
ANOVA FDR = 0.16
0
10
20
30
40
E0P1H0
E0P1H1
E1P0H0
E1P0H1
ER0PR1
ER1PR0
HER0
HER1

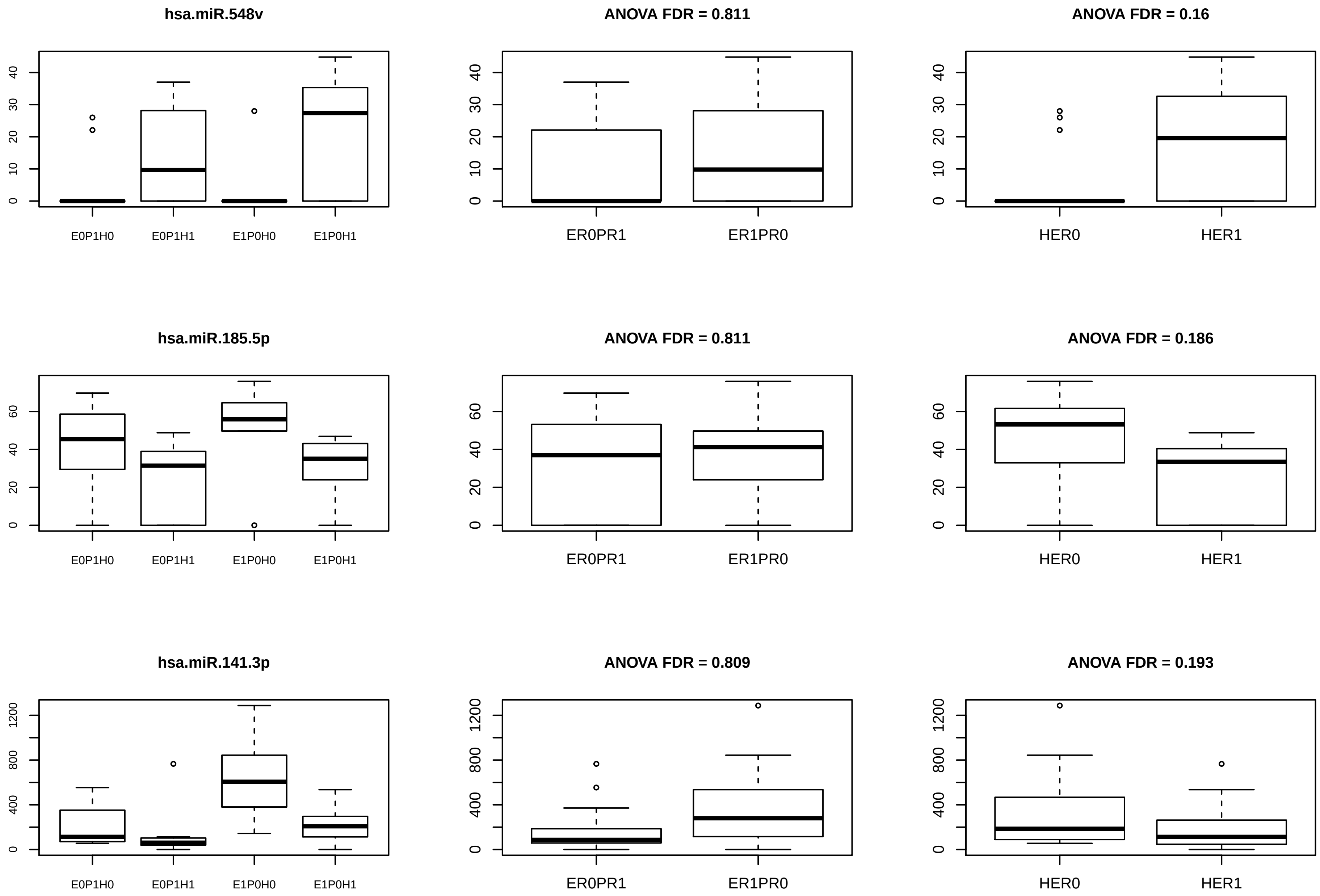
hsa.miR.548v
ANOVA FDR = 0.811
ANOVA FDR = 0.16
E0P1H0
E0P1H1
E1P0H0
E1P0H1
ER0PR1
ER1PR0
HER0
HER1
hsa.miR.185.5p
ANOVA FDR = 0.811
ANOVA FDR = 0.186
hsa.miR.141.3p
ANOVA FDR = 0.809
ANOVA FDR = 0.193
