# Supplementary material for: microRNA Expression Profile in Single Hormone Receptor-Positive Breast Cancers Is Mainly Dependent on HER2 Status—A Pilot Study
Source: Diagnostics (Basel). 2020 Aug 20;10(9):617. doi: 10.3390/diagnostics10090617 (PMC7555149; doi:10.3390/diagnostics10090617)
Supplement: Supplementary file 1 [file diagnostics-10-00617-s001.zip › Sup_Fig_5.pdf]

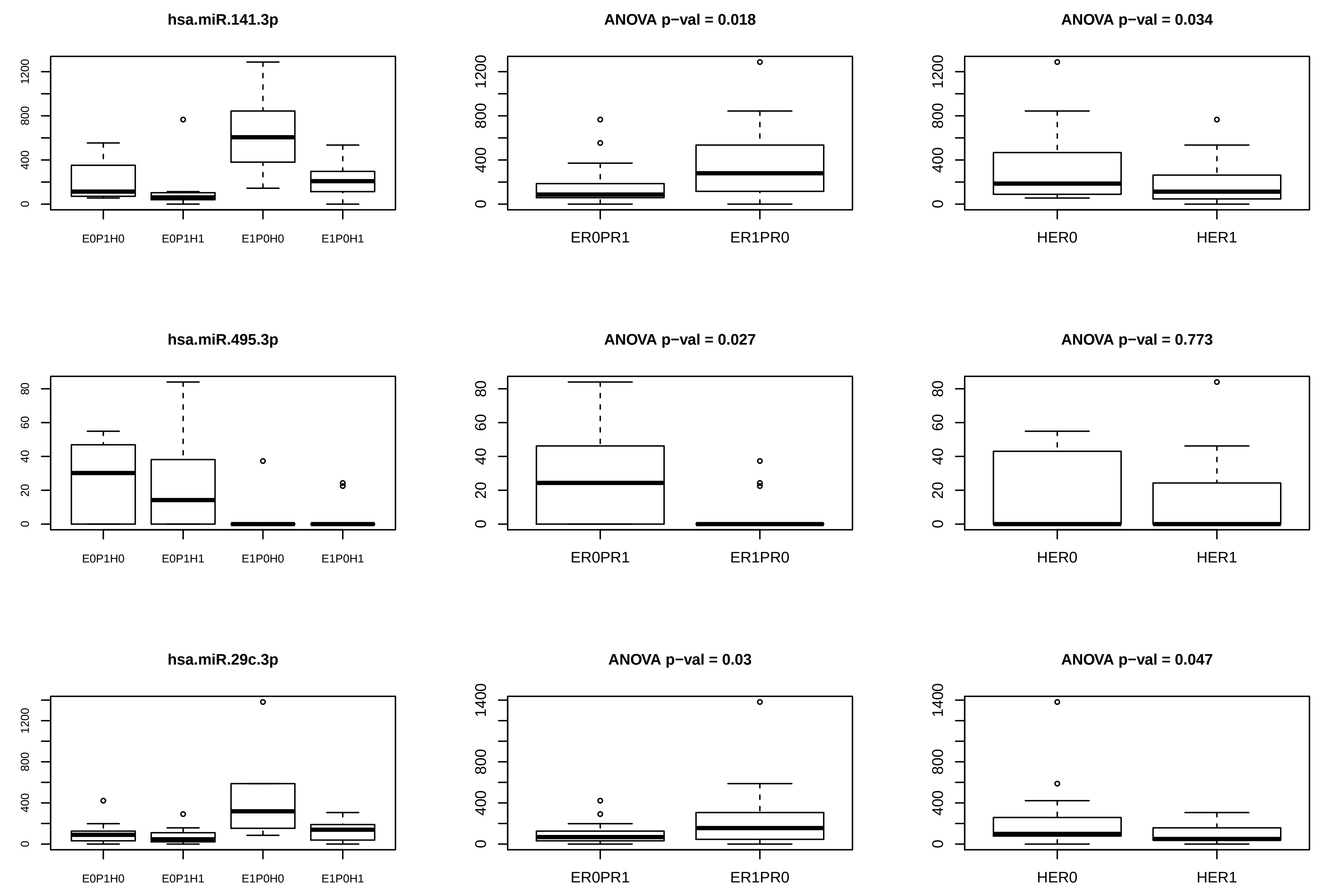
hsa.miR.141.3p
ANOVA p−val = 0.018
ANOVA p−val = 0.034
0
400
800
1200
E0P1H0
E0P1H1
E1P0H0
E1P0H1
ER0PR1
ER1PR0
HER0
HER1
hsa.miR.495.3p
ANOVA p−val = 0.027
ANOVA p−val = 0.773
0
20
40
60
80
E0P1H0
E0P1H1
E1P0H0
E1P0H1
ER0PR1
ER1PR0
HER0
HER1
hsa.miR.29c.3p
ANOVA p−val = 0.03
ANOVA p−val = 0.047
0
400
800
1200
1400
E0P1H0
E0P1H1
E1P0H0
E1P0H1
ER0PR1
ER1PR0
HER0
HER1

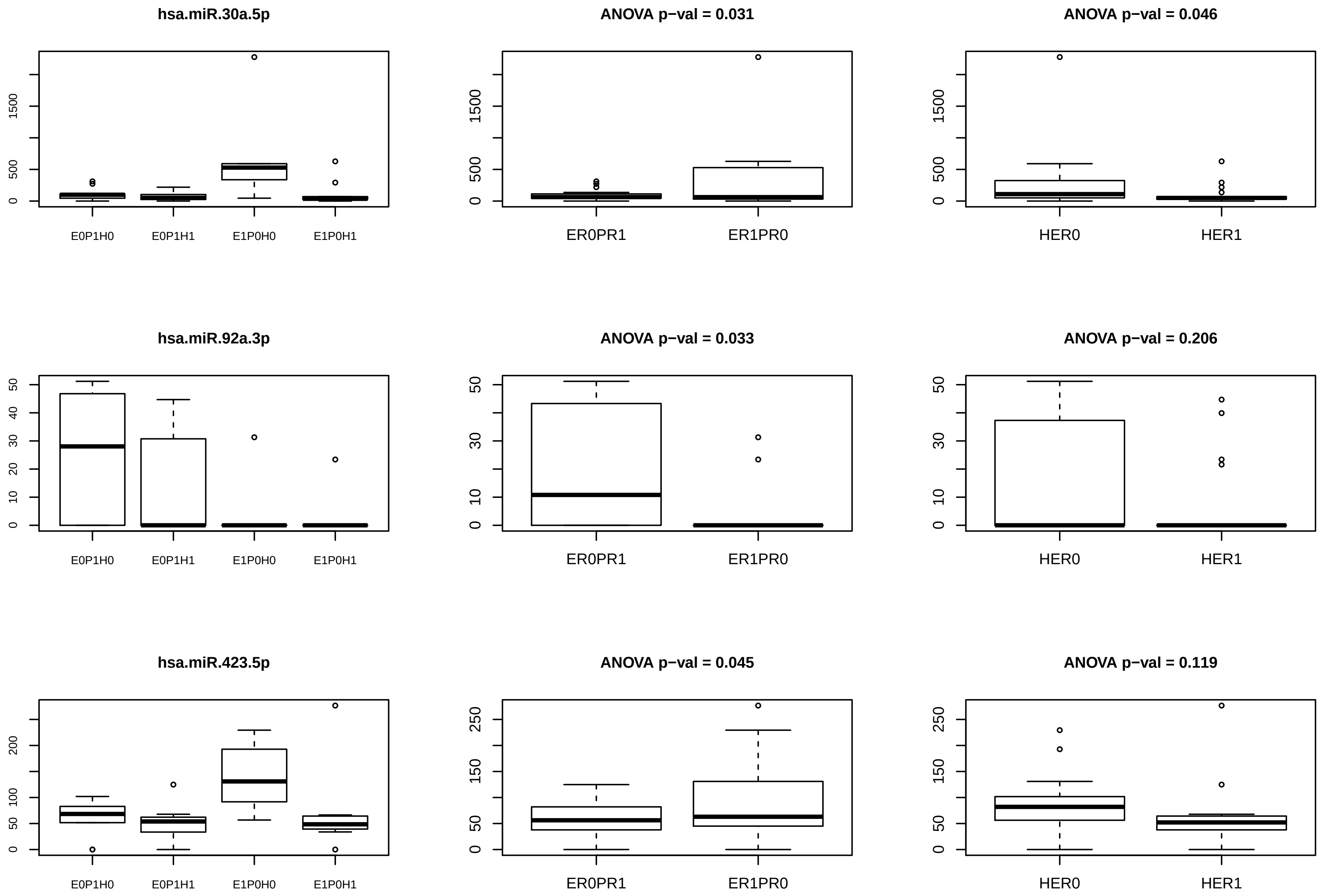
hsa.miR.30a.5p
ANOVA p−val = 0.031
ANOVA p−val = 0.046
0
500
1500
E0P1H0
E0P1H1
E1P0H0
E1P0H1
ER0PR1
ER1PR0
HER0
HER1
hsa.miR.92a.3p
ANOVA p−val = 0.033
ANOVA p−val = 0.206
0
10
20
30
40
50
hsa.miR.423.5p
ANOVA p−val = 0.045
ANOVA p−val = 0.119
0
50
100
150
200
250

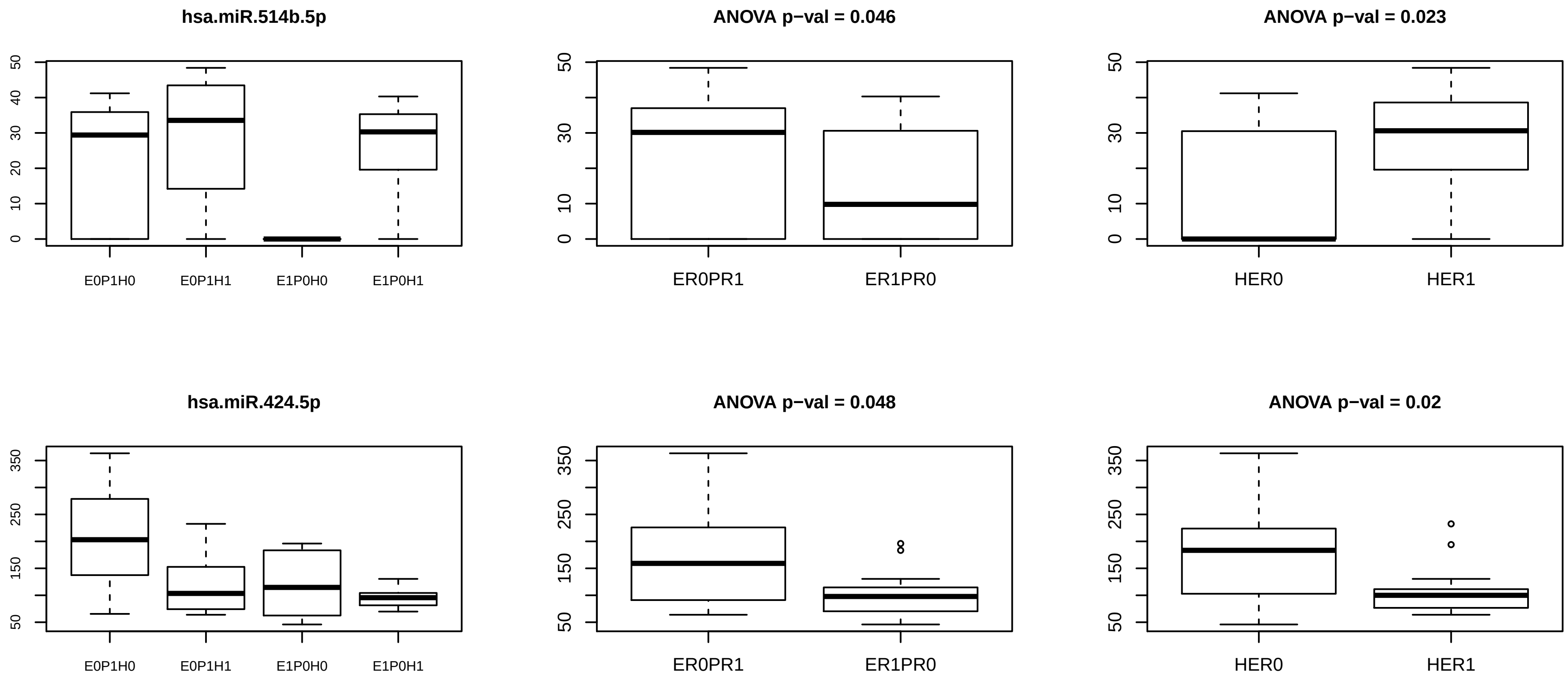
hsa.miR.514b.5p
ANOVA p−val = 0.046
ANOVA p−val = 0.023
0
10
20
30
40
50
E0P1H0
E0P1H1
E1P0H0
E1P0H1
ER0PR1
ER1PR0
HER0
HER1
hsa.miR.424.5p
ANOVA p−val = 0.048
ANOVA p−val = 0.02
50
150
250
350
