# Supplementary figures and images for: microRNA Expression Profile in Single Hormone Receptor-Positive Breast Cancers Is Mainly Dependent on HER2 Status—A Pilot Study
Source: Diagnostics (Basel). 2020 Aug 20;10(9):617. doi: 10.3390/diagnostics10090617 (PMC7555149; doi:10.3390/diagnostics10090617)

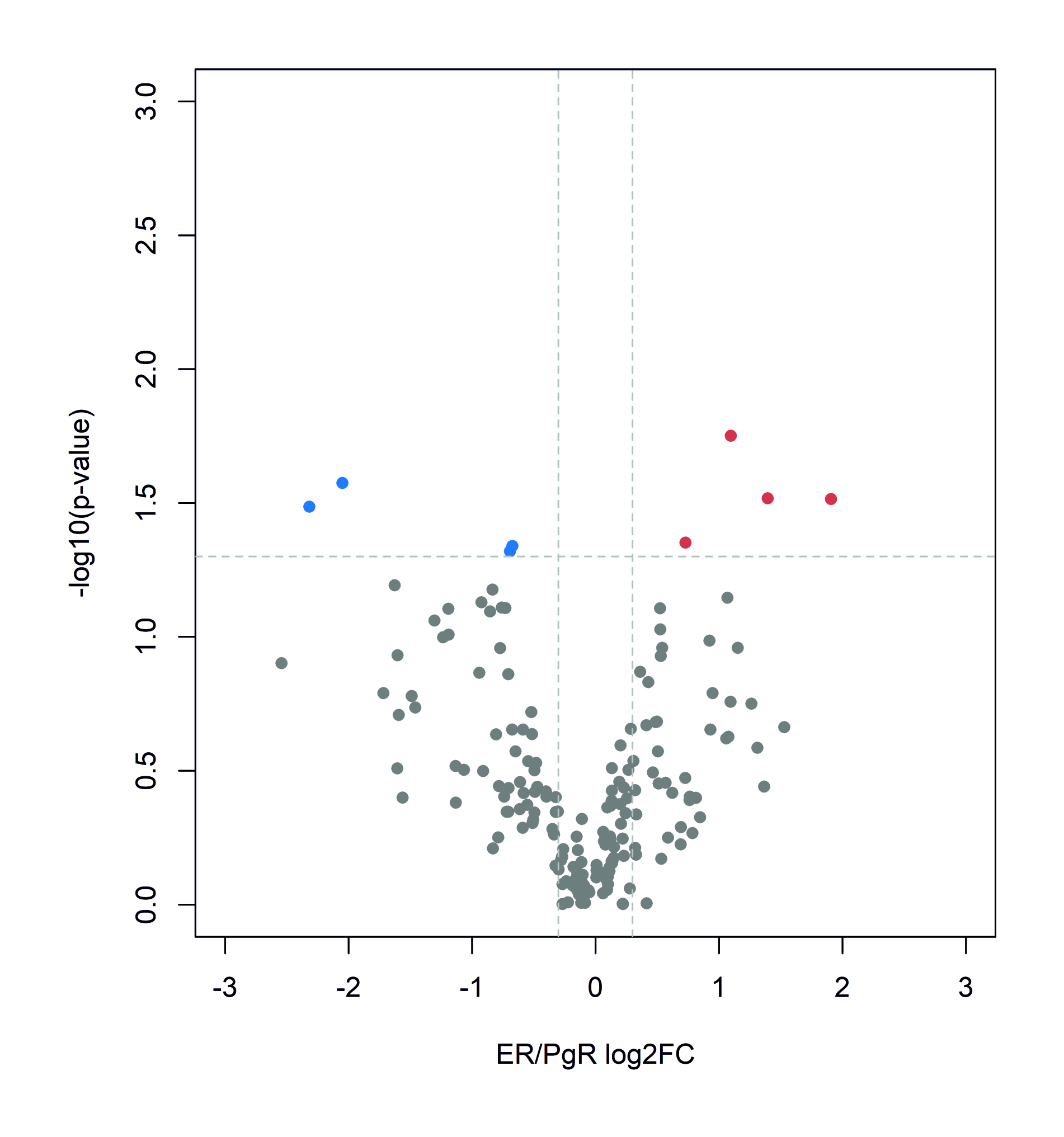

Supplement: Supplementary file 1 [file diagnostics-10-00617-s001.zip › Sup_Fig_6.tif]

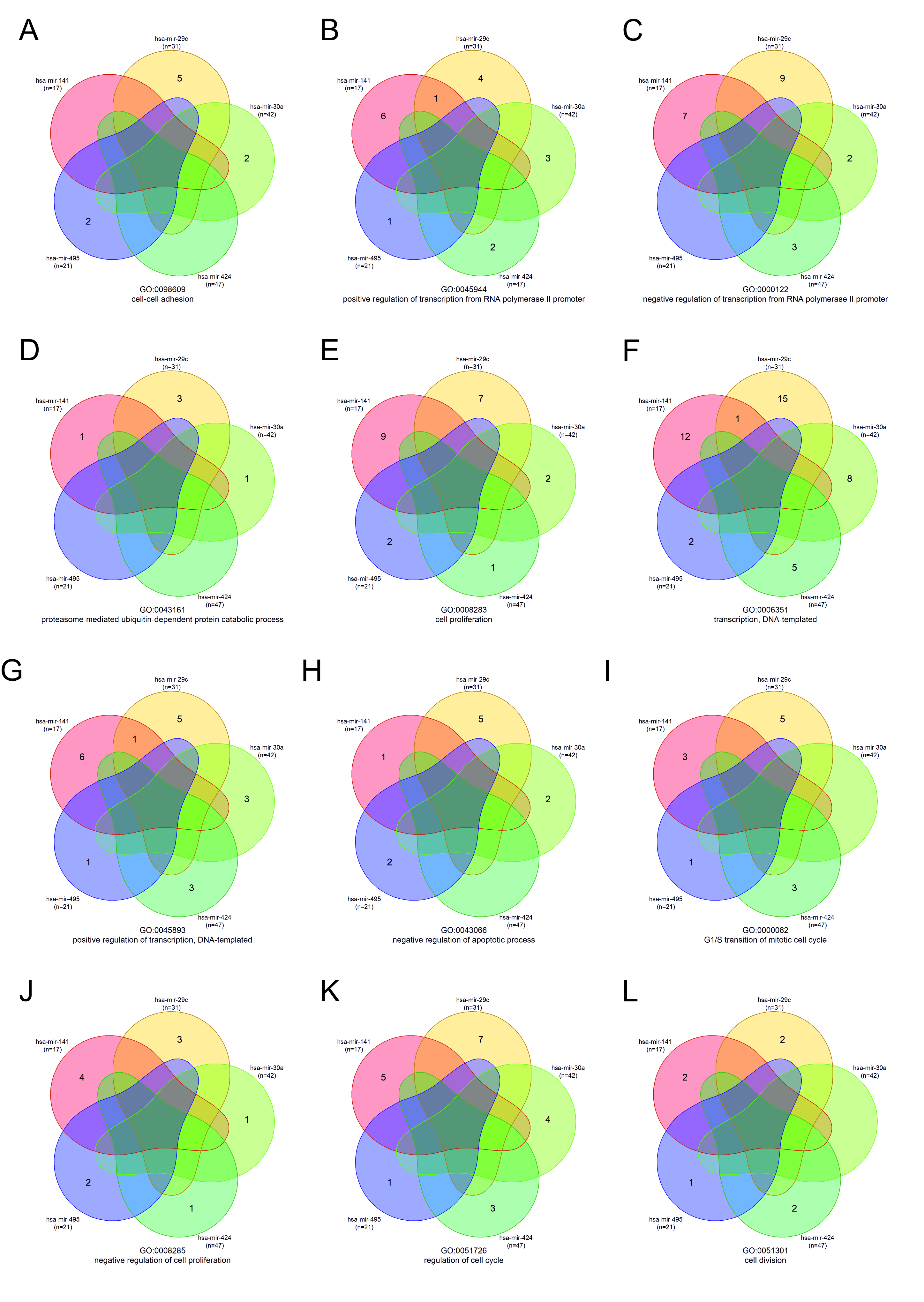

Supplement: Supplementary file 1 [file diagnostics-10-00617-s001.zip › Sup_Fig_7.tif]

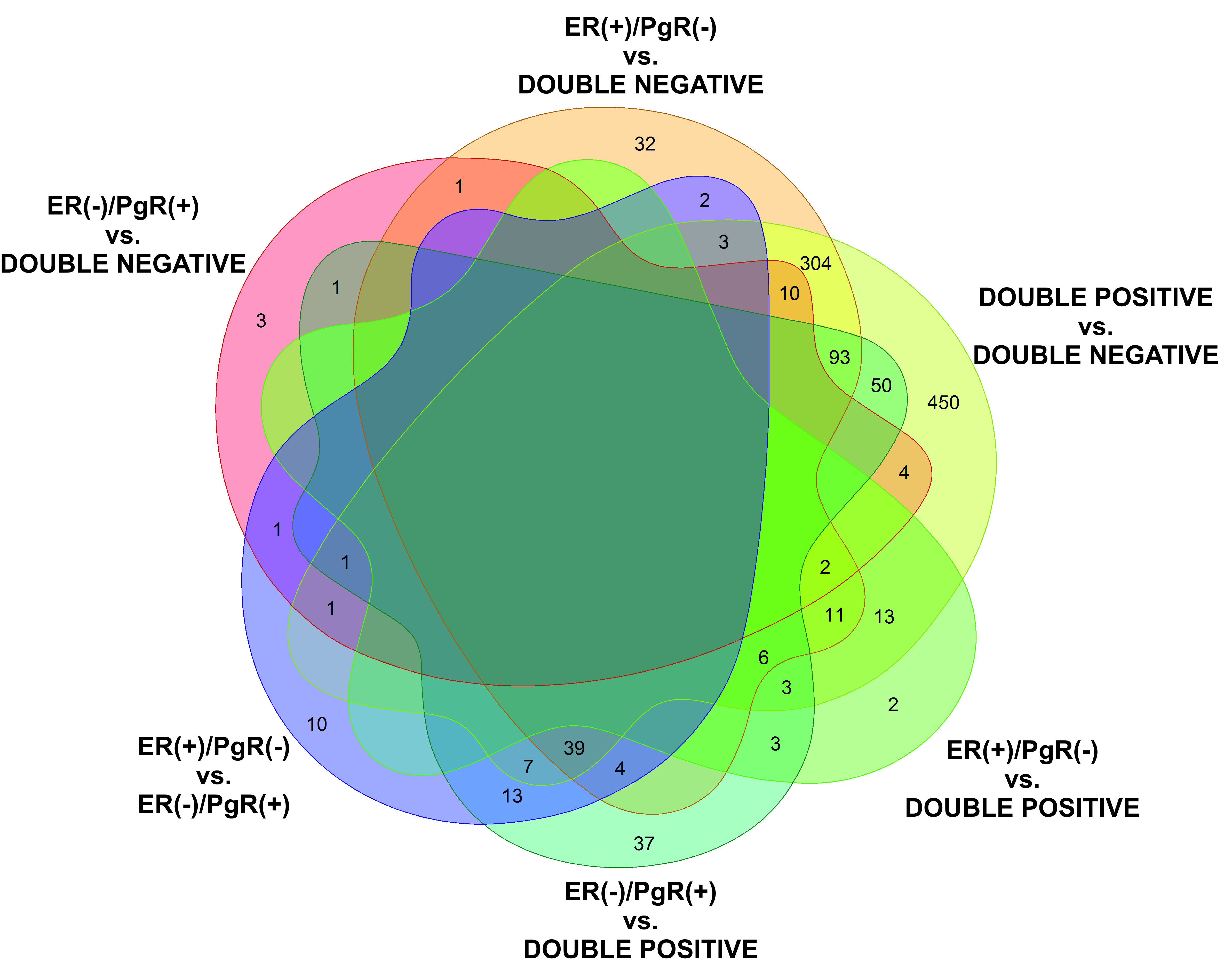

Supplement: Supplementary file 1 [file diagnostics-10-00617-s001.zip › Sup_Fig_8.tif]

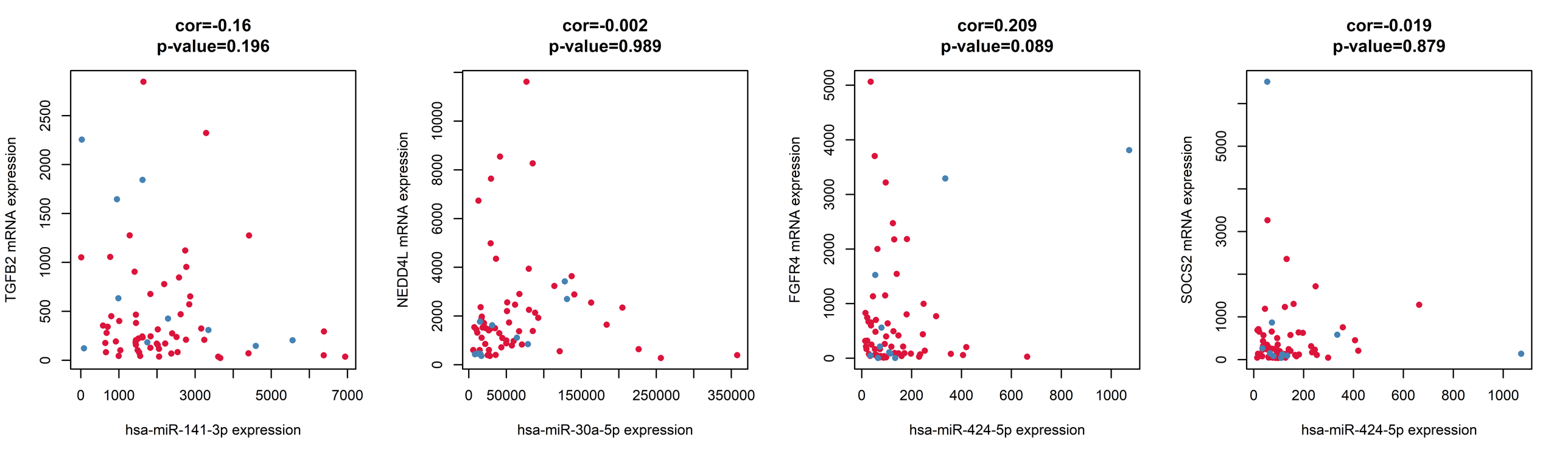

Supplement: Supplementary file 1 [file diagnostics-10-00617-s001.zip › Sup_Fig_9.tif]

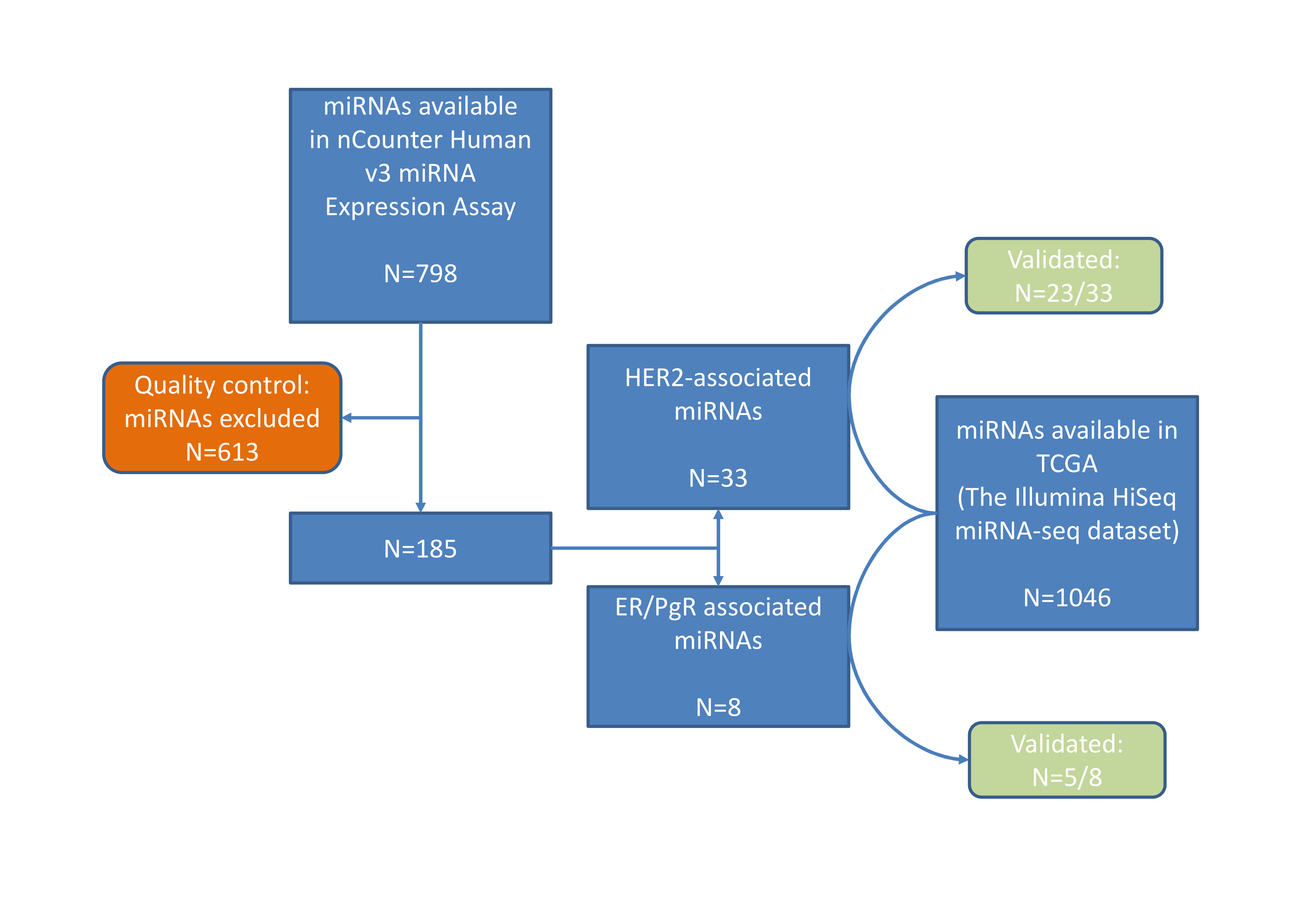

Supplement: Supplementary file 1 [file diagnostics-10-00617-s001.zip › Sup_Fig_1.tif]

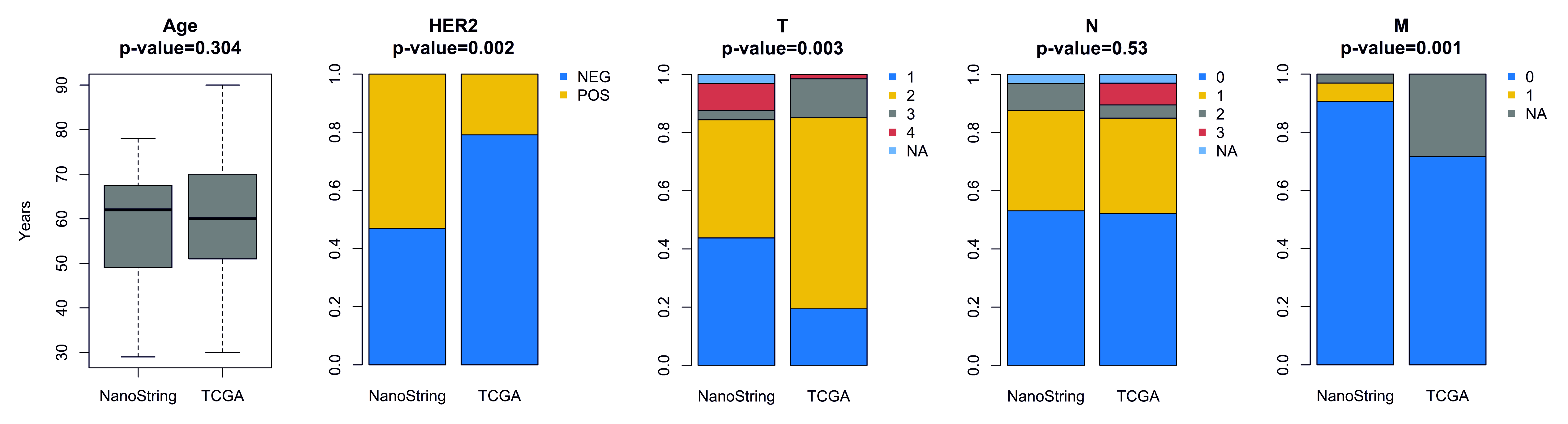

Supplement: Supplementary file 1 [file diagnostics-10-00617-s001.zip › Sup_Fig_2.tif]

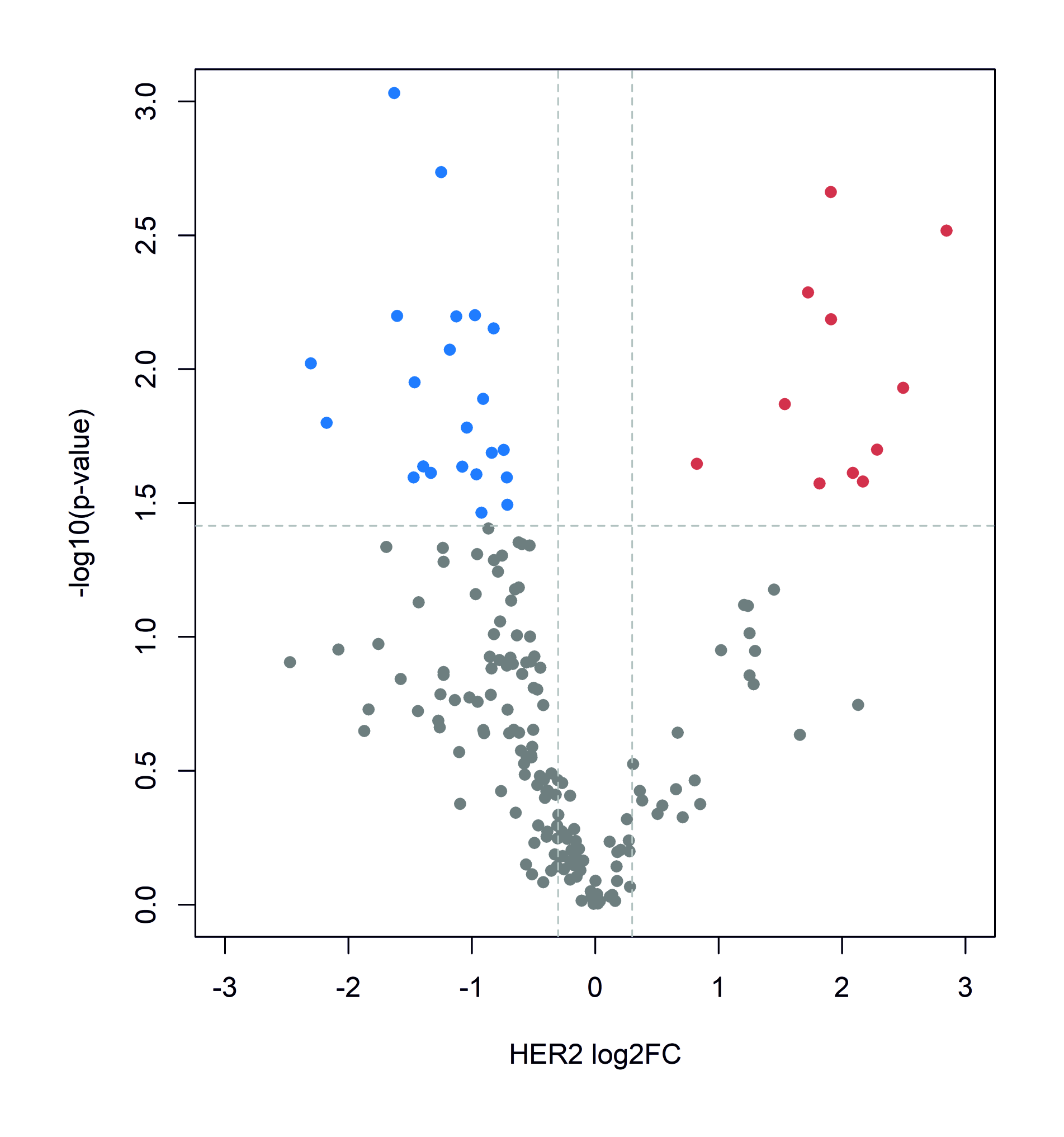

Supplement: Supplementary file 1 [file diagnostics-10-00617-s001.zip › Sup_Fig_4.tif]
